# LA-ICP-MS Zircon U-Pb Ages, geochemical characteristics, and geological significance of the early cretaceous volcanic rocks in Haitangwan Town, Southern Hainan Island, China

**DOI:** 10.1371/journal.pone.0337464

**Published:** 2025-12-04

**Authors:** Yihua Lin, Dingyong Liang, Qinmin Yuan, Zailong Hu, Zhaoying Lv, Changyan Lv

**Affiliations:** 1 Hainan Key Laboratory of Marine Geological Resources and Environment, Haikou, Hainan, China; 2 Hainan Geological Survey, Haikou, Hainan, China; 3 Hainan Institute of Marine Economic Development and Resource Conservation, Haikou, Hainan, China; Ministry of Education, MOROCCO

## Abstract

The Lumuwang Formation in Hainan Island is a key stratum for analyzing Mesozoic tectonic evolution. However, its depositional age relies only on sporopollen evidence, lacks high-precision dating, and its age attribution remains controversial. Additionally, the origin, tectonic setting of its volcanic interlayers and their connection to regional magmatic activities are unclear, which greatly restricts the systematic understanding of Hainan Island’s Mesozoic paleogeography and geotectonic evolution. Targeting a newly discovered set of volcanic rocks hosted in the terrigenous clastic rocks of the Lumuwan Formation in Haitangwan Town, Sanya City, this study systematically conducted zircon U-Pb dating and geochemical analysis. The results show that the minimum age is 121 Ma, thereby accurately constraining the depositional age of the clastic rocks of the Lumuwan Formation to the Early Cretaceous. Geochemical characteristics indicate that the volcanic rocks belong to the high-K calc-alkaline to shoshonitic series. Their low Mg^#^ values and high MF and DI values indicate that the magma underwent highly differentiated evolution. The rare earth element (REE: La-Lu) distribution shows significant enrichment of light rare earth elements (LREE: La-Eu) and strong fractionation between LREE and heavy rare earth elements (HREE: Gd-Lu). Trace elements are characterized by enrichment of large ion lithophile elements (LILE: Rb, Ba, and K) and depletion of high field strength elements (HFSE: Nb, Ta, and Ti), highlighting typical island arc magma attributes. The above geochemical characteristics of the volcanic rocks reveal that the study area was in a transitional tectonic environment from an island arc to back-arc extension during the Early Cretaceous. This provides key empirical evidence for the dynamic process of the transition from a continental margin arc to intracontinental extension around 121 Ma in the South China continental margin, further deepening the scientific understanding of Mesozoic crust-mantle interaction and basin-mountain coupling processes in Hainan Island.

## Introduction

**Fig 1:**
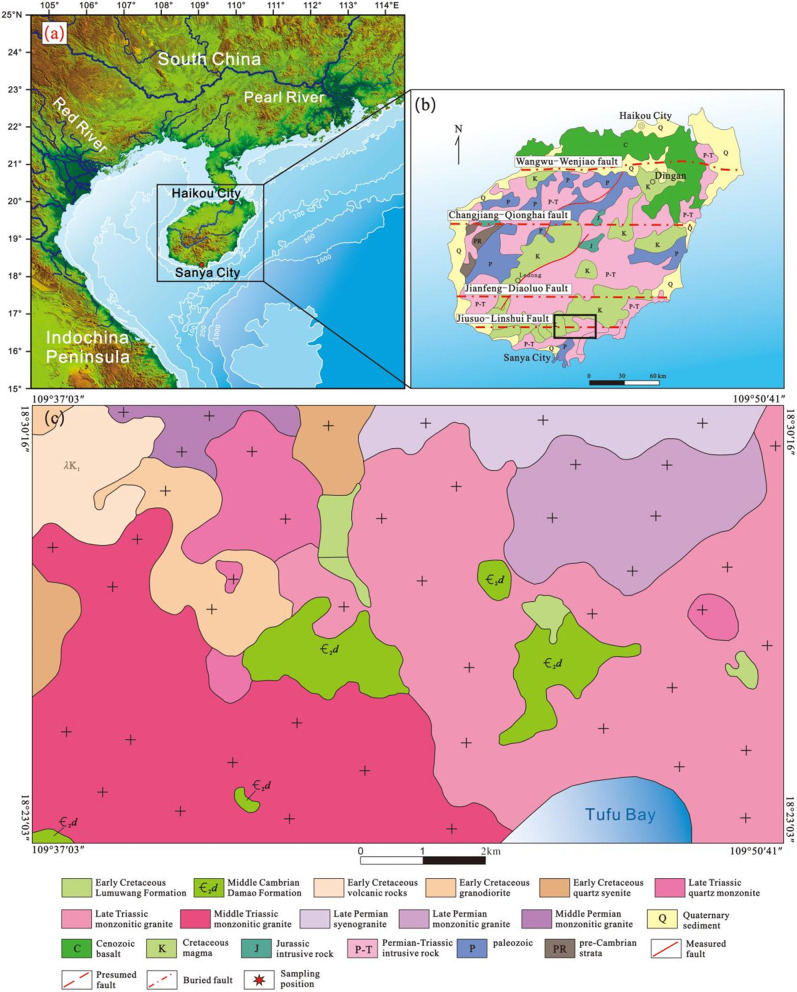
Map of the location of Hainan island. **(a), The base map is adapted from Liang et al. [1], with modifications)、Geological Map of Hainan Island (b) and Study Area (c).** The original basemap was created using the Digital Elevation Model (DEM). The public data of the DEM was obtained from the Geospatial Data Cloud (https://www.gscloud.cn/#page1) and was further processed using software CorelDRAW 2019 version.

Hainan Island is situated at the convergent zone of the Pacific Plate, Eurasian Plate, and Indo-Australian Plate, having undergone a complex geological and tectonic evolution since the Mesozoic. Its unique geotectonic location renders it an ideal region for studying the evolutionary history of continental margins, the process of continental accretion in eastern Asia, and the formation mechanism of the South China Sea (Fig 1a) [2–6]. Early Cretaceous sedimentary basins are widely developed across Hainan Island. These basins have completely recorded the tectonic evolution of the South China Block (SCB) and are of crucial significance for evaluating numerous Pacific subduction models. Previous studies have indicated that the formation of Hainan’s sedimentary basins was a response to regional uplift, generally controlled by a compressional tectonic setting [7,8], which stands in marked contradiction to the understanding that magmatic activities of the same period were driven by lithospheric extension [9]. With the continuous accumulation of high-precision dating data, the academic community’s understanding of the tectonic evolution of the South China margin is constantly deepening [10–12]. Under the constraint of an accurate chronological framework, determining the tectonic attributes of Hainan’s Cretaceous basins is not only the core to unraveling the geodynamic processes of the South China Block [9] but also the key to revealing the dynamic connection between the Paleo-Pacific and Tethys subduction zones [13].

The Early Cretaceous intracontinental extensional tectonic stage in Hainan Island is prominently characterized by the development of a series of extensional fault basins and the emplacement of high-potassium volcanic rocks. Volcanic rocks of this period are widely exposed; apart from a small amount occurring as interlayers in the sedimentary rocks of the Lumuwan Formation, most occur as continental volcanic rock series. The lithostratigraphic units, from bottom to top, can be divided into the Liuluocun Formation, Tangtaling Formation, and Lingkecun Formation, which are mainly distributed along the Jiuxian-Lingshui Fault Zone and its adjacent areas such as Baoting, Ledong, and Sanya, with sporadic outcrops also in Wuzhishan in the central part, as well as Luoji, Wangshang, and Dongfang in northern Hainan [14]. As a typical Cretaceous “red-bed basin”, the Leiming Basin in the Ding’an tectonic area contains strategic mineral resources such as gold, molybdenum, and rare earth ores, thus holding significant research value [15]. Through comparative analysis of sporopollen assemblages from the Lumuwan Formation in the Changpo-Wangwu Basin of western Hainan, Long constrained its age to the middle-late Early Cretaceous [16]; whereas Huang, based on the characteristics of sporopollen assemblages in the Hongmao section of Ledong, assigned the age of the Lumuwan Formation to the Aptian-early Albian [17]. However, the determination of the depositional age of this formation currently relies solely on sporopollen biostratigraphic evidence, lacking constraints from high-precision isotopic dating data.

Approximately 3 km northeast of Haitangwan Town, Sanya City, the authors discovered a new suite of volcanic rock series occurring as interlayers within the terrigenous clastic rocks of the Lumuwan Formation. The rock types are dominated by bedded tuffs and tuffaceous sedimentary rocks, belonging to a typical volcanic-sedimentary facies association. Currently, there remains a lack of systematic research on scientific issues such as the mineral composition, formation age, and petrogenesis of the Cretaceous Lumuwan Formation tuffs. This knowledge gap has significantly hindered the comprehensive analysis of the Mesozoic paleogeographic framework and geotectonic evolution of Hainan Island. In this study, LA-ICP-MS zircon U-Pb isotopic dating was conducted on the Early Cretaceous volcanic rocks from Haitangwan, Sanya, accurately constraining their formation age to the early stage of the Early Cretaceous. Meanwhile, through geochemical analyses of major, trace, and rare earth elements, the petrogenetic type and tectonic setting attributes were thoroughly discussed. The research results are expected to provide new geological constraints for studying the Mesozoic, especially Cretaceous magmatic evolution processes and the formation mechanisms of sedimentary basins in Hainan Island.

### Geological settings

Hainan Island, with its unique geotectonic location, has undergone complex crustal evolution through multiple stages from the Jinning to Yanshanian and Himalayan periods, resulting in major tectonic features dominated by E-W, NE-SW, and N-S trending structures [18]. The NE-SW trending structures are mainly represented by the central Baisha Fault and the Gezhen Fault in western Hainan (Fig 1b). The E-W trending structures include the Jiuxian-Lingshui tectonic belt, Jianfeng-Diaoluo tectonic belt, Wangwu-Wenjiao tectonic belt, Dongfang-Qiongzhong tectonic belt, Changjiang-Qionghai tectonic belt, and Gancheng-Wuzhishan tectonic belt [5,19].

Hainan Island has a complete stratigraphic record; except for the absence of the Devonian System, strata from the Proterozoic to Holocene are all distributed here. The Mesoproterozoic Baoban Group is the oldest known stratum in Hainan Island to date [20]. Magmatic activity in Hainan Island is intense and widespread, characterized by multi-stage emplacement. Variscan-Indosinian granitoids are the most widely distributed magmatic rocks on the island, predominantly composed of monzogranite and granodiorite [2,21,22]. Volcanic activity in Hainan Island has a multi-stage eruptive history: Cenozoic basalts are widely distributed in northern Hainan, while Mesozoic intermediate-acidic volcanic rocks are mainly developed in southern Hainan [23].

After the Variscan orogeny, seawater retreated from the entire island during the middle-late Permian, leaving the crust exposed to long-term denudation. The Indosinian orogeny led to the formation of extensive molasse deposits in piedmont basins, accompanied by frequent orogenic movements and magmatic activity [24]. In the Late Jurassic or earlier, the Pacific Plate subducted beneath the Eurasian Continent, with the pre-subduction tectonic stress being compressive. This intense compressive movement was strongly resisted by the South China Sea platform; to release energy, the Hainan paleoland formed NE-SW trending faulted basins on the pre-Mesozoic basement [25–27].

## Methods

Samples used for zircon U-Pb dating were mainly collected from artificially excavated scarps. The rocks are fresh, with a lithology of rhyolitic lithic-crystal tuff, and a total of 4 samples were collected. Zircon separation was carried out in the laboratory of Hebei Institute of Regional Geological and Mineral Survey. Conventional gravity and magnetic separation methods were used to separate zircon minerals, followed by zircon selection under a binocular microscope. The selected zircons were mounted in epoxy resin, and then ground and polished to expose their surfaces. Cathodoluminescence (CL) microphotography was performed on the zircons to be dated.

Zircon CL imaging and LA-ICP-MS analysis were completed in the State Key Laboratory of Geological Processes and Mineral Resources, China University of Geosciences (Wuhan). CL images were taken using a JEOL-JXA-8100 electron microprobe. Zircon U-Pb isotope and trace element content analysis were conducted using a GeoLas2005 excimer laser ablation system (MicroLas) coupled with an Agilent 7500a ICP-MS instrument (Agilent, USA). For zircon dating, the standard zircon 91500 was used as the external standard, and GJ-1 as the internal standard. For element content determination, SRM610 served as the external standard, and ^29^Si as the internal standard element. The measured isotope data were processed offline using ICPMSDataCal 8.3. Zircon U-Pb concordia diagrams, calculation of weighted mean ages, and graphing were performed using Isoplot 3.0. The error for individual data points is 1σ, and the confidence level for the weighted mean age of samples is 2σ (95% confidence interval).

Whole-rock major element, trace element, and rare earth element (REE) analyses were all conducted at Hubei Institute of Geological Experiments. For major element analysis, H_2_O content was determined by the gravimetric method, CO_2_ by non-aqueous titration, and all other oxides by the X-ray fluorescence (XRF) spectrometry with the α-coefficient method. The analytical precision (relative error) was 1% except for H_2_O. Trace elements and REEs were analyzed by inductively coupled plasma atomic emission spectrometry (ICP-AES). The long-term (4-hour) stability of the instrument was better than 1%, and the analytical precision was better than 1%. Spectral interference was addressed by processing the analyzed peak shapes using a peak formation function, i.e., Fitted background correction [28].

### Results

#### Petrological characteristics.

**Fig 2:**
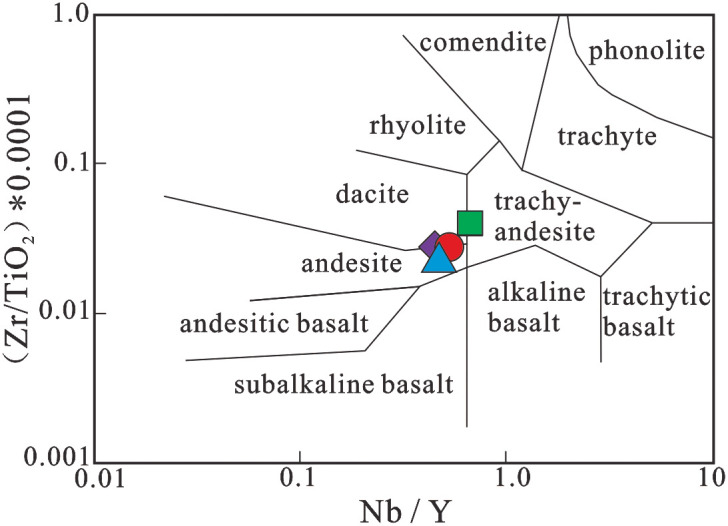
Zr/ TiO_2_-Nb/ Y diagram of volcanicrocks(after Winchester and Floyd [29]).

**Fig 3:**
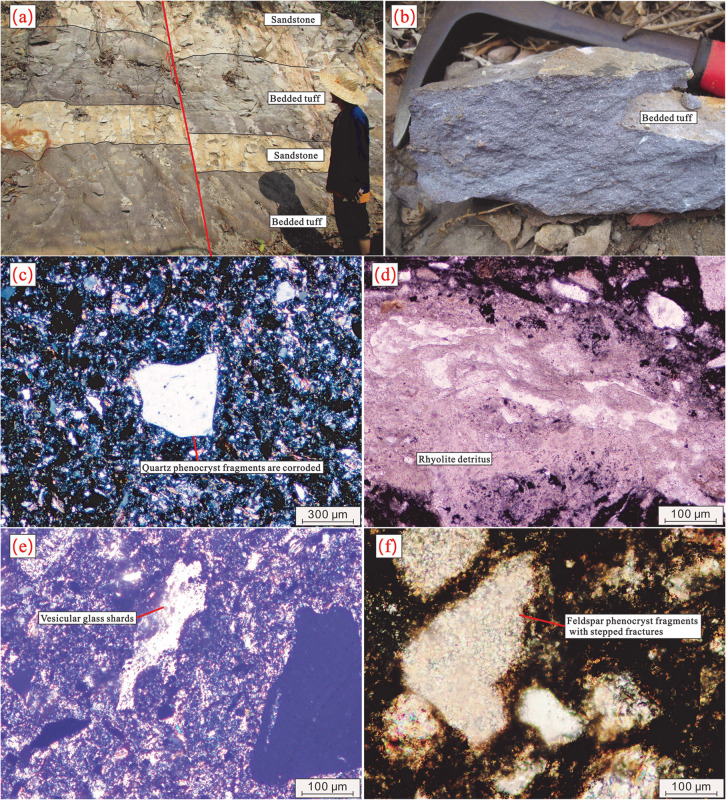
Field and Microscopic Photographs of Tuff. a Interbedded photograph of tuff and sandstone; b Close-up photograph of tuff specimen; c Photograph of corroded quartz phenocrysts, cross-polarized light (XPL); d Photograph of rhyolitic lithic fragments, plane-polarized light (PPL); e Photograph of volcanic glass shards, XPL; f Photograph of feldspar phenocrysts with conchoidal fracture, XPL.

Volcanic rocks are mainly exposed as interlayers within the Early Cretaceous terrigenous clastic rocks of the Lumuwan Formation in the Haitangwan Town area, Sanya City (Fig 1c). They predominantly belong to the volcanic-sedimentary facies, with bedded tuff as the main rock type. On the Zr/TiO_2_–Nb/Y diagram (Fig 2), most of the volcanic rocks plot in the dacite and andesite fields. The characteristics of the exposed rocks are described as follows: The lithology is rhyolitic lithic-crystal tuff (Figs 3a, 3b), exhibiting purple, light purple-red, and gray colors, with a tuffaceous texture and massive structure. The rock is composed of crystal fragments (25%–30%), lithic fragments (5%–10%), and volcanic ash (65%±). Crystal fragments range in size from 0.02 mm to 2.0 mm, with quartz being the dominant component, followed by feldspar. Quartz crystals are mostly angular, while some crystal and lithic fragments are corroded into rounded granular or embayed shapes (Fig 3c). Feldspar crystal fragments are strongly sericitized or kaolinized, showing a torn shape with stepped fractures (Fig 3d). Lithic fragments are plastic, appearing as flowing elongated strips with a width of 0.5 mm–1.5 mm and a length of up to 5 mm, mainly consisting of rhyolite lithics (Fig 3e). The rock also contains a small amount of devitrified acicular-vitreous fragments (Fig 3f). Crystal fragments, lithic fragments, and vitreous fragments are cemented by volcanic ash in a matrix-supported manner.

### LA-ICP-MS zircon U-Pb ages

**Fig 4:**
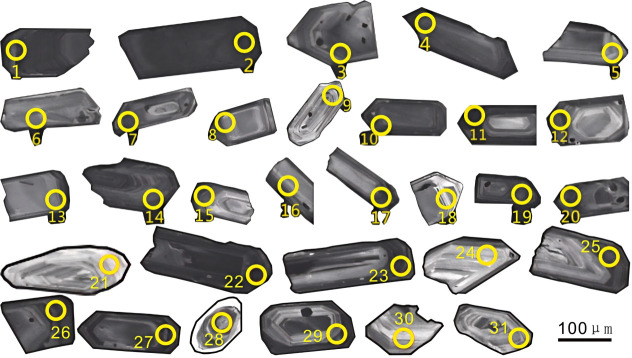
Sample PMD05-15 partial zircon CL image.

The Th/U ratio of zircon can be used to discriminate the genesis of rocks. It is generally accepted that magmatic zircons typically have a Th/U ratio in the range of 0.1–1, whereas those with a Th/U ratio less than 0.1 are of metamorphic origin [30–32]. Most zircons from sample PMD05–15 are euhedral to subhedral prismatic, colorless and transparent, with lengths generally ranging from 100 μm to 210 μm. Cathodoluminescence (CL) images reveal clear rhythmic structures and distinct oscillatory zoning, which are characteristic of magmatic crystallization zircons (Fig 4).

**Fig 5:**
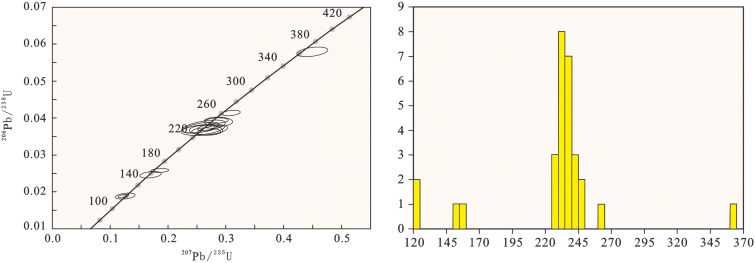
Concordia diagram and histogram of zircon U-Pb ages for sample PMD05-15.

Among the 31 analyzed spots, some zircons show surface dissolution. During LA-ICP-MS analysis, laser ablation spots were positioned to avoid the dissolved areas, mostly at the zircon margins. Two spots with low concordance were excluded, and the remaining ones exhibit good concordance, all lying on or near the concordia line (Fig 5). The zircon U-Pb dating data of the 31 spots are listed in Table 1. The zircons have relatively high Th and U contents: Th contents range from 72.5 ppm to 1500 ppm, with an average of 325.20 ppm; U contents range from 132 ppm to 1937 ppm, with an average of 720.3 ppm. The Th/U ratios vary from 0.207 to 1.019, mostly between 0.3 and 0.8, indicating typical magmatic zircons [33,34].

**Table 1:** Results of Single-Grain Zircon U-Pb analysis for tuff from Haitang Town, Hainan Island.

Spot number	Th (ppm)	U (ppm)	Th/U	^207^Pb/^206^Pb		^207^Pb/^235^U		^206^Pb/^238^U		^207^Pb/^206^Pb		^207^Pb/^235^U		^206^Pb/^238^U	
				Ratio	±1σ	Ratio	±1σ	Ratio	±1σ	Age	±1σ	Age	±1σ	Age	±1σ
01	206	661	0.3112	0.0510	0.0016	0.2651	0.2651	0.0375	0.0004	243	76.8	239	6.6	237	2.4
02	431	1524	0.2825	0.0521	0.0016	0.2722	0.2722	0.0378	0.0004	300	75.0	244	6.5	239	2.2
04	619	636	0.9729	0.0518	0.0016	0.2661	0.2661	0.0371	0.0004	276	67.6	240	6.4	235	2.2
05	72.5	198	0.3659	0.0498	0.0027	0.2553	0.2553	0.0371	0.0005	187	126	231	10.8	235	2.9
07	209	509	0.4101	0.0495	0.0020	0.2589	0.2589	0.0380	0.0004	169	94.4	234	8.1	241	2.6
08	136	254	0.5350	0.0495	0.0022	0.2576	0.2576	0.0377	0.0004	169	136	233	9.2	238	2.6
09	182	345	0.5265	0.0492	0.0022	0.1693	0.1693	0.0247	0.0003	167	104	159	6.8	157	1.8
10	296	680	0.4363	0.0525	0.0015	0.2728	0.2728	0.0376	0.0004	306	63.0	245	6.0	238	2.2
11	401	1937	0.2070	0.0540	0.0011	0.3095	0.3095	0.0415	0.0003	369	48.1	274	4.8	262	2.0
12	436	532	0.8206	0.0527	0.0019	0.2780	0.2780	0.0383	0.0004	317	81.5	249	7.7	242	2.6
13	198	454	0.4360	0.0501	0.0018	0.2617	0.2617	0.0378	0.0004	198	80.5	236	7.6	239	2.3
14	233	734	0.3169	0.0558	0.0013	0.4489	0.4489	0.0580	0.0005	456	51.8	377	7.5	363	3.2
15	180	545	0.3298	0.0521	0.0016	0.2766	0.2766	0.0383	0.0004	300	73.1	248	6.9	243	2.8
16	216	429	0.5044	0.0530	0.0021	0.2840	0.2840	0.0388	0.0005	328	90.7	254	8.7	246	2.8
17	318	781	0.4075	0.0522	0.0018	0.2680	0.2680	0.0369	0.0004	295	79.6	241	7.7	234	2.4
18	113	218	0.5172	0.0513	0.0030	0.2575	0.2575	0.0366	0.0005	254	133	233	11.7	231	3.2
19	237	700	0.3387	0.0515	0.0018	0.2613	0.2613	0.0365	0.0004	261	75.0	236	7.3	231	2.5
20	319	943	0.3381	0.0497	0.0015	0.2551	0.2551	0.0370	0.0004	189	75.0	231	6.3	234	2.3
21	111	153	0.7282	0.0491	0.0032	0.1257	0.1257	0.0190	0.0003	154	144	120	6.3	121	1.9
22	649	1683	0.3853	0.0496	0.0010	0.2482	0.2482	0.0361	0.0002	176	46.3	225	4.0	229	1.4
23	580	1317	0.4404	0.0513	0.0011	0.2666	0.2666	0.0375	0.0003	257	50.0	240	4.7	237	1.8
24	95.1	132	0.7182	0.0532	0.0029	0.2690	0.2690	0.0371	0.0005	345	124	242	10.8	235	3.0
25	330	976	0.3386	0.0505	0.0012	0.2539	0.2539	0.0362	0.0002	217	84.2	230	4.7	229	1.6
26	301	1008	0.2990	0.0502	0.0012	0.2520	0.2520	0.0362	0.0003	211	55.5	228	4.6	230	1.6
27	140	431	0.3253	0.0520	0.0015	0.2823	0.2823	0.0392	0.0004	283	66.7	252	6.5	248	2.5
28	1500	1472	1.0191	0.0531	0.0012	0.2801	0.2801	0.0379	0.0003	345	50.0	251	5.1	240	1.9
29	593	980	0.6057	0.0468	0.0014	0.1224	0.1224	0.0190	0.0002	35.3	74.1	117	3.3	121	1.0
30	94.3	187	0.5059	0.0496	0.0022	0.2508	0.2508	0.0369	0.0004	176	99.1	227	8.7	234	2.7
31	235	470	0.5002	0.0519	0.0017	0.1855	0.1855	0.0258	0.0002	280	77.8	173	5.3	164	1.5

The ^20^^6^Pb/^238^U ages of the 29 spots range from 121 Ma to 363 Ma. Among them, the 121 Ma ages of two zircons fall on the concordia line, which can represent the eruption and crystallization age of the tuff in Haitangwan Town, Sanya City. The ages of the remaining zircons are mainly concentrated around 236 Ma. The occurrence of these Triassic inherited zircon cores first indicates that the formation of the Early Cretaceous volcanic rocks is closely related to the involvement of ancient crustal materials. During the Triassic (~250–200 Ma), Hainan Island and the South China region were in a post-Indosinian orogeny tectonic adjustment stage, accompanied by large-scale granitic magmatism and regional metamorphism, which led to the formation of an ancient crustal basement dominated by Triassic granites and metamorphic rocks [9,10,12]. During the Early Cretaceous, under the influence of the retreat of the Pacific Plate subduction or the regional extensional tectonic setting, partial melting or assimilation-contamination of the ancient lower crustal rocks was triggered. The Triassic zircons were not completely dissolved and were incorporated into the Early Cretaceous magma in the form of “inherited cores”, eventually forming the zircon core-mantle structure observed today.

### Petrogeochemical characteristics

A total of four samples were collected from the Early Cretaceous volcanic rocks in the study area for petrochemical analysis. The analytical results, CIPW normative mineral compositions, and petrochemical parameters are presented in Table 2. After correcting for loss on ignition (LOI), the analytical data were recalculated to 100% anhydrous basis. The SiO₂ contents range from 66.62% to 74.47%, classifying these rocks as acidic. CIPW norm calculations indicate a dominant mineral assemblage of Q + Or + Ab + Di + Hy + C, with quartz (Q) accounting for 43.99%–58.89%, orthoclase (Or) 20.98%–28.00%, and minor corundum. All samples show zero content of anorthite (An), with accessory minerals including apatite (Ap), ilmenite (Ilm), and magnetite (Mt). Compared to the average chemical composition of global rhyodacites, these volcanic rocks are characterized by relatively low contents of CaO, MgO, and Na_2_O, and elevated contents of Fe_2_O_3_, Al_2_O_3_, and K_2_O.

**Table 2:** Major element compositions, CIPW normative mineral contents, and common petrochemical parameters of Early Cretaceous volcanic rocks in the study area.

Sample name	Number	Lithology			Chemical composition analysis (wt%)									
					SiO_2_	TiO_2_	Al_2_O_3_	Fe_2_O_3_	MnO	MgO	CaO	Na_2_O	K_2_O	P_2_O_5_
PMD05–15	1	Rhyolitic lithic-crystal tuff			63.99	0.80	18.35	7.06	0.02	0.80	0.07	0.39	4.50	0.09
D2510-1	2				71.76	0.57	15.74	4.33	0.02	0.31	0.01	0.16	3.41	0.04
D2510-3A	3				66.27	0.79	17.42	6.87	0.00	0.39	0.02	0.26	4.49	0.10
D2510-3B	4				66.36	0.80	17.37	6.90	0.00	0.40	0.02	0.25	4.56	0.10
CIPW normative mineral contents (%)														
Loss on ignition(LOI)	Total	Q	An	Ab	Or	Ne	C	Di	Hy	Il	Mt	Ap	Alkali feldspar	Plagioclase
3.29	99.36	43.99	0	3.4	27.83	0	13.43	0	4.94	1.6	4.66	0.21	31.23	0
3.40	99.75	58.89	0	1.44	20.98	0	12.25	0	2.41	1.12	2.86	0.09	22.42	0
3.06	99.68	47.64	0	2.29	27.53	0	12.61	0	3.68	1.56	4.55	0.23	29.82	0
3.06	99.81	47.39	0	2.21	28	0	12.46	0	3.66	1.56	4.58	0.23	30.21	0
Common petrochemical parameters														
Mg^#^	AIK	TFe	FeO*/MgO		A/CNK		A/NK	DI	SI	A.R	σ	τ	A/MF	C/MF
22.25	4.89	6.35	7.99		3.26		3.33	75.22	6.46	1.72	1.09	22.38	1.67	0.01
15.31	3.57	3.9	12.44		3.954		3.98	81.31	3.87	1.59	0.44	27.23	2.5	0
12.54	4.75	6.18	15.77		3.276		3.29	77.46	3.41	1.75	0.94	21.67	1.78	0
12.77	4.81	6.21	15.56		3.221		3.25	77.6	3.38	1.77	0.97	21.50	1.77	0

Note: Mg^#^ = 100×(MgO/40.4)/(MgO/40.4 + 0.0098*Fe_2_O_3_^T^); AIK = K_2_O+Na_2_O; AKI=(K_2_O+Na_2_O)/Al_2_O_3_; A/CNK = Al_2_O_3_/(CaO + Na_2_O+K_2_O); A/NK = Al_2_O_3_/(Na_2_O+K_2_O); DI = Q(Quartz)+Or(Orthoclase)+Ab(Albite)+Ne(Nepheline)+Kf(K-feldspar); SI= (Ol(Olivine)+Hy(Hypersthene)+Di(Diopside)+Tr(Tremolite)+Mt(Magnetite)+Il(Ilmenite))/Total×100; A.R = Na_2_O+K_2_O/(SiO_2_-Al_2_O_3_-TiO_2_); σ= (Na_2_O+K_2_O)^2^/(SiO_2_ − 43); τ=(Na_2_O+K_2_O)/(FeO + MgO); A/MF = Al_2_O_3_/(MgO + FeO); C/MF = CaO/(MgO + FeO)

**Fig 6:**
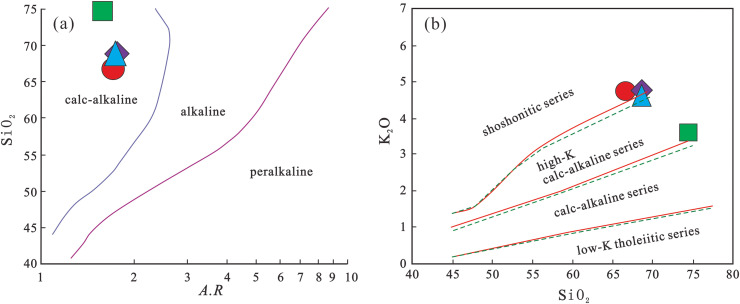
SiO_2_-A. **R diagram (after Wright [35]) and SiO**_**2**_ **-K**_**2**_ **O diagram (after Rickwood [36]) for volcanic rocks in the study area.**

The Early Cretaceous volcanic rocks exhibit pronounced peraluminous affinity, as evidenced by high Al_2_O_3_ contents and A/CNK values ranging from 3.221 to 3.954. All four samples yield A/CNK > 1.1 and A/NK values between 3.25 and 3.98, combined with the presence of corundum in the CIPW norm, indicating a strongly peraluminous series. The alkali ratio (A.R) values range from 1.59 to 1.77, plotting in the calc-alkaline field on the SiO_2_-A.R diagram (Fig 6a). The silica-alkali diagram (Fig 2) further confirms their subalkaline nature. The Rittmann index (σ) values of 0.44–1.09 classify these rocks as typical calc-alkaline series. On the SiO_2_-K_2_O diagram (Fig 6b), all samples plot within the high-K calc-alkaline to shoshonitic fields, with K_2_O > Na_2_O - 2, indicative of potassic affinity. The CIPW norm Q values of 43.99%–58.89% suggest highly silica-saturated magma.

Petrochemical parameters suggest extensive magmatic differentiation: Mg^#^ values range from 12.54 to 22.25, solidification index (SI) from 3.38 to 6.46, differentiation index (DI) from 75.22 to 81.31, and maficity index (MF) from 89.87 to 94.53. The coupling of high DI with low SI, along with elevated MF values, collectively indicates advanced fractional crystallization.

### Rare earth element geochemical characteristics of rocks

**Fig 7:**
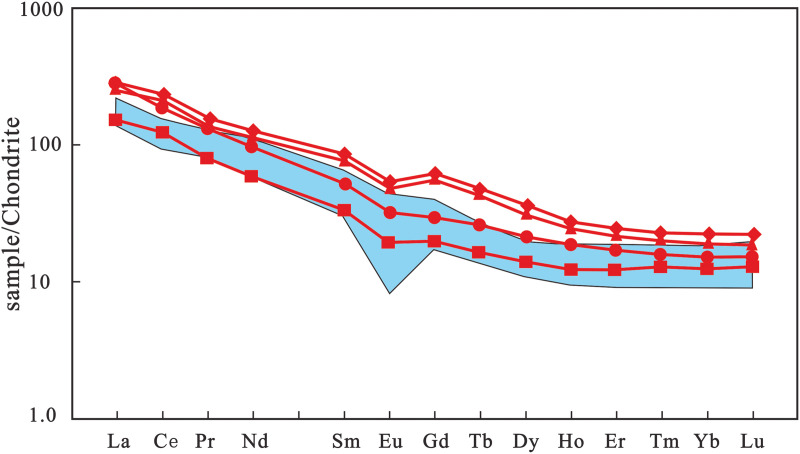
Chondrite-normalized REE distribution pattern diagram of volcanic rocks in the study area. (Shaded areas represent the background values of Mesozoic volcanic rocks in Hainan Island [14]; chondrite standard values after Sun and McDonough [39]).

Studies have shown that ratios such as (La/Sm)_N_ and (La/Yb)_N_ are valuable geochemical proxies for examining the degree of LREE to MREE and LREE to HREE segregation during the evolution of different geological systems, respectively [37,38]. Analytical results of rare earth elements (REEs) for four samples of Early Cretaceous volcanic rocks in the study area are presented in Table 3. The total REE contents of the rock samples vary slightly, ranging from 163.69 × 10^−6^ to 326.66 × 10^−6^. The ratios of light rare earth elements to heavy rare earth elements (LREE/HREE) are between 8.73 and 12.51, with (La/Yb)_N_ values of 12.14–17.96 and (Ce/Yb)_N_ values of 9.65–12.56. There is no significant negative Eu anomaly, as indicated by δEu values of 0.72–0.81, suggesting that the source region might have undergone partial melting of plagioclase-free rocks or that plagioclase hardly fractionated during magma crystallization. The δCe values range from 0.94 to 1.07, showing no anomalous variation in Ce. The (La/Sm)_N_ values are 3.19–5.32, and (Gd/Yb)_N_ values are 1.56–2.89, indicating strong fractionation of LREEs but insignificant fractionation of HREEs. On the chondrite-normalized REE distribution diagram (Fig 7), the four samples exhibit similar REE distribution patterns, generally characterized by right-inclined curves with LREE enrichment and relatively flat curves in the HREE segment.

**Table 3:** Summary of REE contents and key characteristic parameters of Early Cretaceous volcanic rocks in the study area.

Sample name	Lithology	REE contents (ppm)															
		La	Ce		Pr	Nd	Sm	Eu		Gd	Tb	Dy		Ho	Er	Tm	Yb
PMD05–15	Rhyolitic lithic-crystal tuff	63.3	114		12.5	44.2	7.68	1.85		5.93	0.97	5.49		1.02	2.80	0.39	2.53
D2510-1		35.8	73.5		7.56	27.2	4.96	1.08		3.99	0.61	3.48		0.68	2.01	0.32	2.11
D2510-3A		63.3	135		14.2	56.3	12.4	2.93		12.1	1.70	8.51		1.47	3.75	0.56	3.46
D2510-3B		65.1	140		14.5	57.7	13.2	3.09		12.5	1.78	9.06		1.53	4.04	0.56	3.62
Key geochemical parameters																	
Lu	ΣREE	LREE		HREE		L/H	(La/Yb)_N_		(Ce/Yb)_N_		(La/Sm)_N_		(Gd/Yb)_N_		δEu	δCe	La/Sm
0.37	263.48	243.98		19.50		12.51	17.96		12.56		5.32		1.94		0.81	0.94	8.24
0.32	163.69	150.17		13.52		11.11	12.14		9.65		4.66		1.56		0.72	1.04	7.22
0.50	316.57	284.54		32.03		8.88	13.14		10.87		3.29		2.89		0.72	1.06	5.09
0.53	326.66	293.08		33.58		8.73	12.90		10.69		3.19		2.85		0.73	1.07	4.95

Note: N denotes values normalized to chondrite.; δEu = Eu_N_/(Sm_N_ × Gd_N_)^1/2^; δCe = 2 × Ce_N_/(La_N_ + Pr_N_); The raw data are available in S1 Appendix.

### Trace element geochemical characteristics of rocks

**Fig 8:**
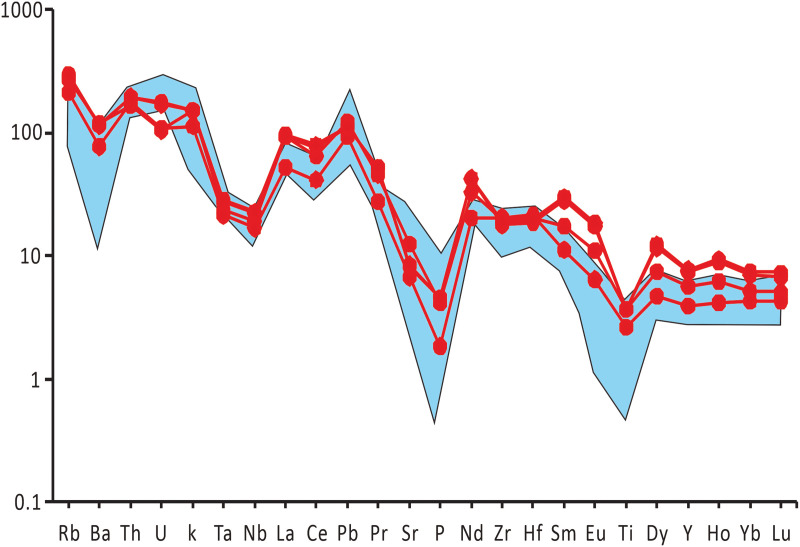
Trace element ratio spidergram of volcanic rocks in the study area. (Shaded areas represent the background values of Mesozoic volcanic rocks in Hainan Island [14]; primitive mantle standard values after Sun and McDonough [39]).

Analytical results of trace elements for four samples of Early Cretaceous volcanic rocks in the study area are presented in Table 4. Their trace element contents are comparable to the average values of Vogt’s acid rocks, with slightly lower contents of Li, Be, Zn, Ta, etc., and slightly higher contents of Sc, V, Hf, Tl, etc., while the remaining elements are close to the average contents of Vogt’s acid rocks [40]. As shown in Table 4, the high-field-strength elements (HFSEs) Zr and Hf in the Early Cretaceous volcanic rocks of the study area have relatively high contents, with Zr ranging from 198 × 10^−6^ to 229 × 10^−6^ and Hf from 5.76 × 10^−6^ to 6.61 × 10^−6^. In contrast, Nb and Ta have low contents, with Nb varying from 11.9 × 10^−6^ to 16.1 × 10^−6^ and Ta from 0.87 × 10^−6^ to 1.15 × 10^−6^. The Nb/Ta ratios range from 13.93 to 14.14, which are lower than those of chondrite, primitive mantle (Nb/Ta = 17.4 ± 0.5), and depleted mantle (Nb/Ta = 15.5 ± 1.0), slightly higher than that of continental crust (Nb/Ta = 11–12), and fall between the depleted mantle and continental crust. Large ion lithophile elements (LILEs) are significantly enriched: Rb contents range from 134 × 10^−6^ to 187 × 10^−6^, Sr from 141 × 10^−6^ to 263 × 10^−6^, with Rb/Sr ratios of 0.64–1.04; Ba contents are 535 × 10^−6^–826 × 10^−6^, U is 2.18 × 10^−6^–3.68 × 10^−6^, and Th is 14.0 × 10^−6^–16.6 × 10^−6^. The trace element spidergram patterns of the four samples are basically consistent (Fig 8), mainly characterized by stronger enrichment of highly incompatible elements such as Th, Ba, Rb, and K compared to moderately incompatible elements like Nb, P, Sr, and Zr. Among them, Rb, Th, K, La, Pb, Nd, and Sm show obvious positive anomalies, while Ta, Ba, P, Sr, Nb, Ce, and Ti exhibit distinct negative anomalies, with a notable feature of TNT negative anomalies. These characteristics are highly similar to those of granites formed in the tectonic setting of normal continental arcs in orogenic belts.

**Table 4:** Table of trace element contents of Early Cretaceous volcanic rocks in the study area.

Sample name			Number		Lithology					Trace element contents (ppm)																							
										Li			Be		Sc			V			Cr		Co		Ni			Cu			Zn		Ga
PMD05–15			1		Rhyolitic lithic-crystal tuff					12.9			1.90		13.9			119			39.8		7.46		14.2			16.1			61.7		20.1
D2510-1			2							3.88			1.62		7.84			74.0			30.3		2.21		5.43			9.34			11.6		16.7
D2510-3A			3							4.70			2.16		14.5			107			33.8		1.06		2.83			8.31			11.1		21.7
D2510-3B			4							4.82			2.18		14.8			109			35.2		1.07		2.78			8.68			11.6		22.3
Trace element contents(ppm)																																	
Rb	Sr			Y		Zr		Nb			Sn			Cs			Ba			Hf		Ta		Tl			Pb			Th		U	
169	263			25.7		220		13.6			2.39			4.21			826			6.29		0.96		1.08			22.6			14.0		2.18	
134	141			17.8		229		11.9			2.15			4.81			535			6.61		0.87		0.86			17.2			15.1		2.27	
177	173			33.4		198		15.7			2.73			6.17			792			5.76		1.12		1.21			20.4			16.4		3.55	
187	179			35.2		211		16.1			2.75			6.43			815			6.13		1.15		1.16			20.8			16.6		3.68	
Characteristic ratios																																	
Nb/Ta		Rb/Sr			K/Rb		Sr/Ba		Zr/Hf			Ti/V				U/Th			Zr/Nb				Ba/Nb			Ba/Th			Rb/Nb			Th/Nb	
14.14		0.64			231.05		0.32		34.95			42.15				0.155			16.158				60.714			58.848			12.39			1.032	
13.57		0.95			219.75		0.26		34.62			47.79				0.15			19.314				45.105			35.31			11.28			1.277	
13.93		1.02			217.52		0.22		34.34			45.77				0.216			12.622				50.543			48.278			11.3			1.047	
13.97		1.04			209.07		0.22		34.36			45.23				0.222			13.094				50.636			49.176			11.64			1.03	

## Discussion

### Formation age of lumuwan formation

The paleontological chronology research on the “red basins” of the Lumuwan Formation in Hainan Island is relatively mature, among which the chronological and biostratigraphic studies of the Yangjiang Red Basin are the most systematic. Long et al. [16], Huang et al. [17,41], and Hainan Institute of Geological Survey [14] have successively collected abundant sporopollen fossils in this region, dominated by gymnosperm sporopollen assemblages, followed by pteridophyte spores. Huang et al. [17] identified a sporopollen assemblage characterized by *Cicatricosisporites*-*Schizaeoisporites*-*Ephedripites*-*Exesipollenites* in the Nanmei area of Ledong County, southern Hainan. Long et al. [16] discovered a sporopollen assemblage represented by *Toroisporis* sp., *Classopollis annulatus*, *Exesipollenites*, *Araucariacites*, and *Palaeoconiferus* sp. in the Lumuwan Formation of the Changpo-Wangwu Basin in Danzhou City. Among the plant fossils collected by Hainan Institute of Geological Survey [14] from Yuanbei, north bank of Longjiang in Qionghai, *Cladophlebis* cf. *exiliformis* is a common taxon in the Late Jurassic to Early Cretaceous strata of China and Japan, while *Cupressionocladus* cf. *gracilis* is mostly found in the Late Jurassic to Early Cretaceous strata of China.

In this study, dating was performed on the intercalated tuffs of the Lumuwan Formation exposed in Haitangwan Town, Sanya City, yielding an eruption-crystallization age of 121 Ma. Based on this, the depositional age of the Lumuwan Formation is clearly defined as the Early Cretaceous. Zhao et al. constrained the depositional duration of the Lumuwan Formation to 109.9–113.3 Ma through zircon U-Pb dating of clastic sedimentary rocks of the Lumuwan Formation and granodiorites intruding them in the Leiming Basin of Ding’an County, northern Hainan Island [42]. Chen et al. analyzed the ages of basic dikes in the Lumuwan Formation in the Ledong area of Hainan Island, indicating that the main body of this formation was deposited in a back-arc extensional basin with an age of 108 Ma [11]. Meng et al. conducted research on the Cretaceous strata in the Baisha Basin and determined the formation age of the Lumuwan Formation in the Ledong area as 106.6 ± 0.3 Ma based on zircon U-Pb dating results of intercalated tuffs in the formation [12].

The 121 Ma age obtained in this study is 8–15 Ma earlier than the aforementioned results, and two possible explanations are proposed: first, multiple phases of volcanic activity occurred during the deposition of the Lumuwan Formation. The tuff dated in this study occurs in the bottom horizon of the formation, whereas sampling locations in other studies may not be from the bottom interval, resulting in age differences. Second, the sedimentary units involved in different studies belong to distinct Cretaceous lake basins. In the context of back-arc extensional tectonics, a series of basins were formed in Hainan Island and its surrounding areas [3,43]. The sampling area of this study is located in southern Hainan Island, where the sedimentary basin formed earliest; thus, the age obtained in this study can better reflect the initial formation age of the Lumuwan Formation. Based on comprehensive analysis, this study prefers the second hypothesis.

### Tectonic setting and geotectonic significance

The Mesozoic volcanic rock belt in the coastal area of Southeast China is located at the southeastern margin of the Eurasian Plate and the southwestern end of the circum-Pacific tectono-magmatic belt. Since the Triassic, affected by the combined action of the circum-Pacific tectonic domain and the Tethys tectonic domain, the magmatic-tectonic activities in this region have shown complex and intense characteristics [21,44–46]. The volcanic rocks in Hainan Island are situated at the southwestern margin of the Mesozoic volcanic rock belt in the coastal area of Southeast China. The Mesozoic tectonic movements here were centered on block faulting activities, which not only triggered the reactivation of ancient tectonic belts but also were characterized by the prominent development of NNE-NE trending structures. This led to frequent magmatic activities in the island during the Late Mesozoic and the formation of zonally distributed red sedimentary basins. During the Early Mesozoic, the tectonic evolution of Hainan Island was mainly controlled by the Tethys tectonic domain [47]. By the Late Mesozoic, the back-arc expansion caused by the subduction of the Pacific Plate brought the block faulting activities in Hainan Island to their peak. A series of high mountains, intracontinental volcanic basins, and sedimentary basins were successively formed during this process [3,23,43,48–50].

Currently, there are two main perspectives regarding the tectonic setting of the Lumuwan Formation: some scholars argue that the deposition of the Lumuwan Formation was a response to the regional uplift of Hainan Island, and it was generally formed in a compressional tectonic environment related to the subduction of the Neo-Tethys [7,51,52]; other scholars propose that this formation was deposited in a rift basin [11,53,54]. This study indicates that Hainan Island was in a critical stage of transition from a continental marginal arc to a rift extensional environment at approximately 121 Ma.

**Fig 9:**
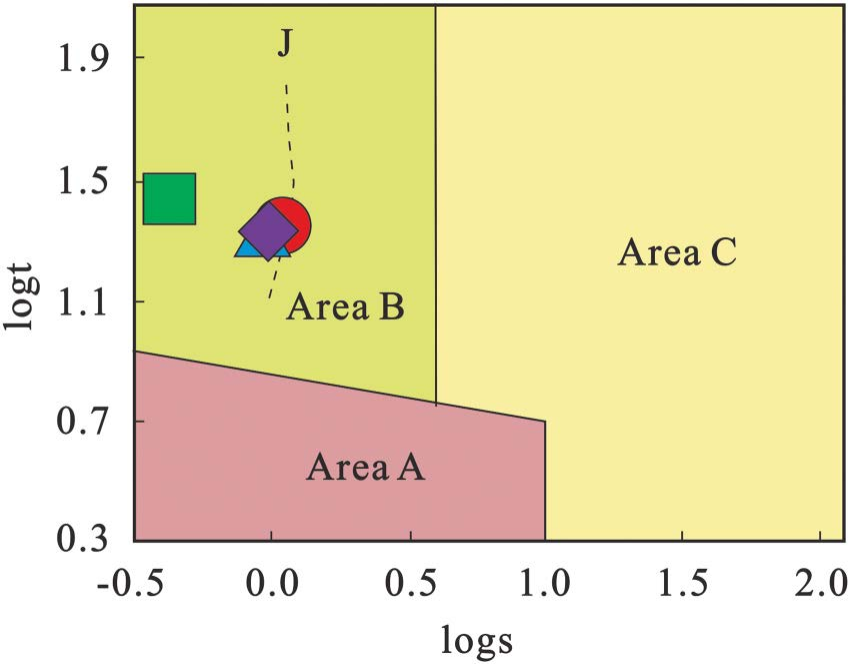
logt-logs diagram of volcanic rocks (after Gottini [55]). Area A: Volcanic rocks from non-tectonic belts (stable intraplate tectonic regions); Area B: Volcanic rocks from orogenic belts (island arcs and active continental margin regions); Area C: Alkaline rocks derived from volcanic rocks in Areas A and B.

**Fig 10:**
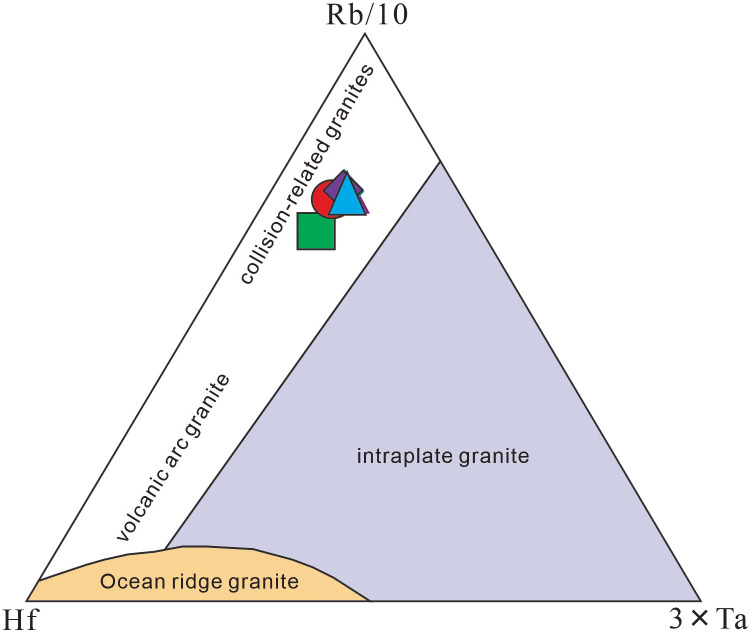
Diagram of Early Cretaceous volcanic rocks Rb-Hf-Ta (after Harris et al. [56]).

**Fig 11:**
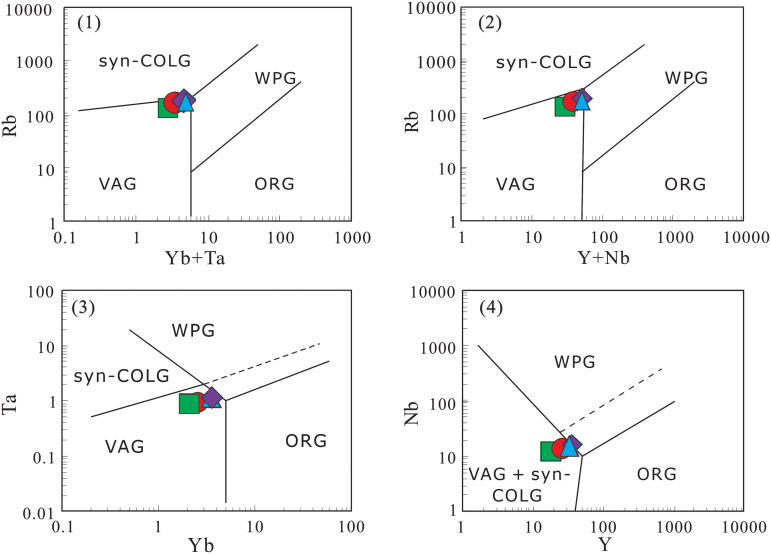
Discriminant diagram of volcanic rocks based on immobile elements (after Pearce et al. [57]). VAG: Volcanic arc granite; WPG: Within-plate granite; S-COLG: Syn-collisional granite; ORG: Ocean ridge granite; A-ORG: Anomalous ocean ridge granite.

**Fig 12:**
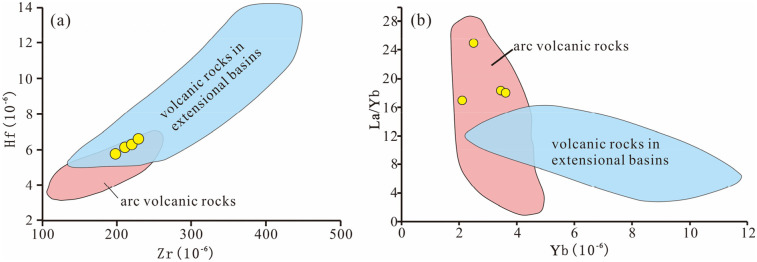
Discriminant diagram of volcanic rock tectonic settings (after Condie [58]).

Geochemical diagram analyses show that: all projection points of the four volcanic rock samples in the Rittmann-Gottardi diagram fall into the areas of island arc, active continental margin and the alkaline volcanic rocks derived therefrom [55], belonging to volcanic rocks formed in an orogenic environment (Fig. 9); in the Rb-Hf-Ta diagram (Fig 10), the projection points are mainly concentrated in the granite area corresponding to the collisional tectonic setting; according to the discriminant diagram of volcanic rock tectonic setting using immobile elements proposed by Pearce et al. [57] (Fig 11), the projection points mainly fall into the volcanic arc tectonic setting area. Additionally, both the Zr-Hf diagram and La/Yb-Yb diagram indicate that the tuffs were formed in a volcanic arc environment (Fig 12).

**Fig 13:**
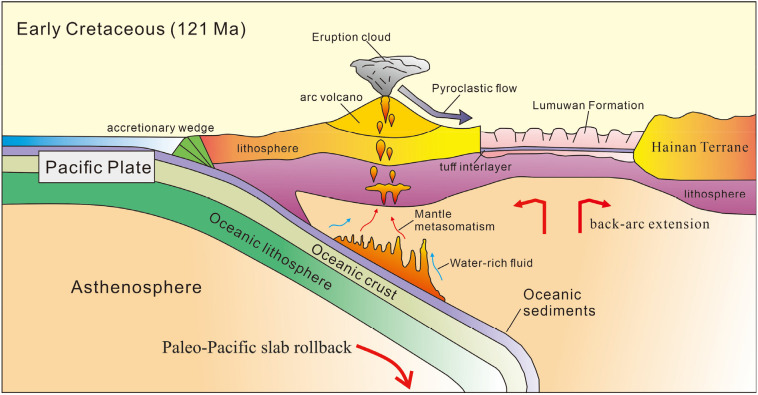
Tectonic evolution model of the study area during the Early Cretaceous.

The Cretaceous tuffs in southern Hainan are not the sole evidence for the existence of a continental marginal arc in this region. The andesites, rhyolites, and granites in the adjacent Lower Cretaceous Liuluocun Formation, Tangtadaling Formation, and Lingkecun Formation all exhibit typical continental marginal arc characteristics [24,59,60]. The tuffs in Haitangwan Town, southern Hainan Island, are mainly distributed near the arc volcanic magmatic activity area. Their magma formation may be closely related to arc magma, suggesting that their source region might have incorporated deep-sea sediments or crustal materials. It is inferred that they were formed by equilibrium reactions of silica-rich melts generated by partial melting of the continental crust and subducted oceanic crust slabs (or sediments) (Fig 13).

Notably, although the aforementioned diagrams indicate a volcanic arc tectonic setting, the projection points are close to the intraplate granite area, showing a tendency of transition to an intraplate tectonic environment. In addition, the Ti/V ratios are lower than the typical range of island arc volcanic rocks (50–100) but close to those of continental rift or intraplate environments (30–50), indicating that the magma formed in an extensional rather than compressional tectonic setting. The Ba/Nb ratios (45.1–60.7) are much higher than those of oceanic island arcs and mid-ocean ridge basalts, approaching the values of continental crust (30–80). The relatively low Sr/Ba ratios, a feature usually associated with plagioclase crystallization or fluid leaching, are common in magmatic differentiation processes in continental extensional environments. Contemporaneous granitic magmatism was intense and multi-stage, dominated by Jurassic and Cretaceous granites. The magma intruded along multi-directional faults, and the rocks lack directional fabrics, indicating that the magmas of this period were products of crust-mantle magma mixing in an extensional tectonic setting [9,44,61].

It has been proposed by some scholars that crustal extension along the South China continental margin initiated at approximately 136 Ma [9,62]. However, based on the data from this study, the tectonic environment was still in a transitional stage at 121 Ma. Stable crustal extension along the South China continental margin should have commenced around 120 Ma, and reached a stable intraplate extensional setting at 108–93 Ma (marked by mafic dikes) and 102 Ma (marked by bimodal volcanic rocks) [12]. There are significant differences in the overall structure and lithological assemblage of the Lumuwan Formation in Hainan Island: the Lumuwan Formation in the Baisha Basin and Qionghai Basin, studied by Chen et al. [23] and Meng et al. [12], formed between 108 and 113.3 Ma, exhibiting typical characteristics of rift lacustrine basins with a fining-upward sequence [14], which is highly consistent with the sedimentary features of extensional-strike-slip rift basins in southwestern Tibet [63]. In contrast, the Lumuwan Formation in the Haitangwan Basin involved in this study, formed at 121 Ma, consists of feldspathic quartz fine sandstones in the lower part and sandy conglomerates in the upper part, showing a coarsening-upward sequence characteristic of a proximal foredeep environment [63]. This lateral variation indicates that although extensional and strike-slip tectonics constituted the overall tectonic framework in Hainan Island during the early Early Cretaceous, a compressional tectonic setting still existed at the plate margin. Such tectonic differentiation resulted in obvious differences in formation age, sedimentary environment, and geotectonic setting between the lacustrine basins in southern and central Hainan Island.

In summary, the study area was in a tectonic setting characterized by the continuous subduction of the Pacific Plate beneath the Eurasian Plate during the early Early Cretaceous (Fig 13). The rollback of the Paleo-Pacific slab drove the extensional stretching of Hainan Island. This slab rollback allowed the asthenospheric mantle to influx into the mantle wedge, metasomatize the lithosphere via slab-derived fluids, and promote magma generation. Such high-angle subduction created a complex tectonic stress field in the South China region: in the back-arc regions far from the subduction zone, significant extensional stretching occurred, driven by mechanisms such as lithospheric mantle delamination or asthenospheric upwelling, leading to intense reactivation of early fault zones including the Changjiang-Qionghai and Jiusuo-Lingshui fault zones. Back-arc extension at the continental margin provided the dynamic conditions for large-scale magmatism and basin formation [3,43]. For example, the geochemical characteristics of Early Cretaceous igneous rocks in Hainan Island clearly record the magma origin and evolutionary processes related to the back-arc extensional environment [9,12,46,48,64]. With the intensification of extensional rifting, a series of basins formed in Hainan Island and its surrounding areas, accumulating thick sedimentary strata such as sandstones and mudstones. Abundant terrigenous clastics were deposited in these basins, forming Cretaceous “red basins” (red clastic rock formations). Meanwhile, deep and large faults connected the crust and upper mantle, triggering volcanic eruptions and forming intracontinental volcanic-sedimentary rock series basins [59,60,65]. By the late Early Cretaceous, back-arc expansion induced by subduction reached its peak, characterized by large-scale intermediate-acidic volcanic eruptions [11,66]. Tectonic deformation during this stage was complex: normal faults related to extension developed (Fig 13) [53], while local compression and fold structures formed due to plate interactions [14], with extensional tectonics dominating overall [67].

## Conclusions

The rhyolitic lithic-crystal tuffs in Xiegui Village, Haitangwan Town, Sanya City, occur as interlayers within the terrigenous clastic rocks of the Lumuwan Formation. LA-ICP-MS zircon U-Pb dating of 29 valid analyses yields ^206^Pb/^238^U ages ranging from 121 to 363 Ma, with the youngest age of 121 Ma interpreted as the eruption-crystallization age of the tuffs. This age constrains the depositional age of the Lumuwan Formation to the Early Cretaceous. Volcanic rocks from Xiegui Village are dominated by acidic lithologies, belonging to the high-K calc-alkaline to shoshonitic series. Geochemically, they exhibit significant enrichment in light rare earth elements (LREE), pronounced enrichment in large ion lithophile elements (LILE; e.g., Rb, Sr, Ba, K), and pronounced depletion in high field strength elements (HFSE; e.g., Ta, Nb, Ti). Hainan Island was under an extensional tectonic regime during the Early Cretaceous. Episodic mantle upwelling triggered basaltic magma underplating, which induced partial melting of middle-lower crustal materials. By the late Early Cretaceous, the back-arc spreading driven by the rollback/high-angle subduction of the Paleo-Pacific slab reached its acme, triggering large-scale volcanic eruptions. The volcanic rocks in the study area are genetically related to arc magmatism, with their magma sources likely incorporating deep-sea sediments or crustal components.

## Supporting information

S1 Appendix Geochemical raw data. (XLSX)

S1 Fig Fig 1. Map of the Location of Hainan Island (a) Geological Map of Hainan Island (b) and Study Area (c). (DOCX)

S2 Fig Fig 2. Zr TiO_2_-Nb Y diagram of volcanicrocks. (DOCX)

S3 Fig Fig 3. Field and Microscopic Photographs of Tuff. (DOCX)

S4 Fig Fig 4. Sample PMD05–15 partial zircon CL image. (DOCX)

S5 Fig Fig 5. Concordia diagram and histogram of zircon U-Pb ages for sample PMD05–15. (DOCX)

S6 Fig Fig 6. SiO_2_-A. R diagram and SiO_2_-K_2_O diagram.
(DOCX)

S7 Fig Fig 7. Chondrite-normalized REE distribution pattern diagram of volcanic rocks in the study area. (DOCX)

S8 Fig Fig 8. Trace element ratio spidergram of volcanic rocks in the study area. (DOCX)

S9 Fig Fig 9. logt-logs diagram of volcanic rocks. (DOCX)

S10 Fig Fig 10. Diagram of Early Cretaceous volcanic rocks Rb-Hf-Ta. (DOCX)

S11 Fig Fig 11. Discriminant diagram of volcanic rocks based on immobile elements. (DOCX)

S12 Fig Fig 12. Discriminant diagram of volcanic rock tectonic settings. (DOCX)

S13 Fig Fig 13. Tectonic evolution model of the study area during the Early Cretaceous. (DOCX)
