# Supplementary material for: LA-ICP-MS Zircon U-Pb Ages, geochemical characteristics, and geological significance of the early cretaceous volcanic rocks in Haitangwan Town, Southern Hainan Island, China
Source: PLoS One. 2025 Dec 4;20(12):e0337464. doi: 10.1371/journal.pone.0337464 (PMC12677543; doi:10.1371/journal.pone.0337464)
Supplement: S1 Fig — (DOCX) [file pone.0337464.s002.docx]

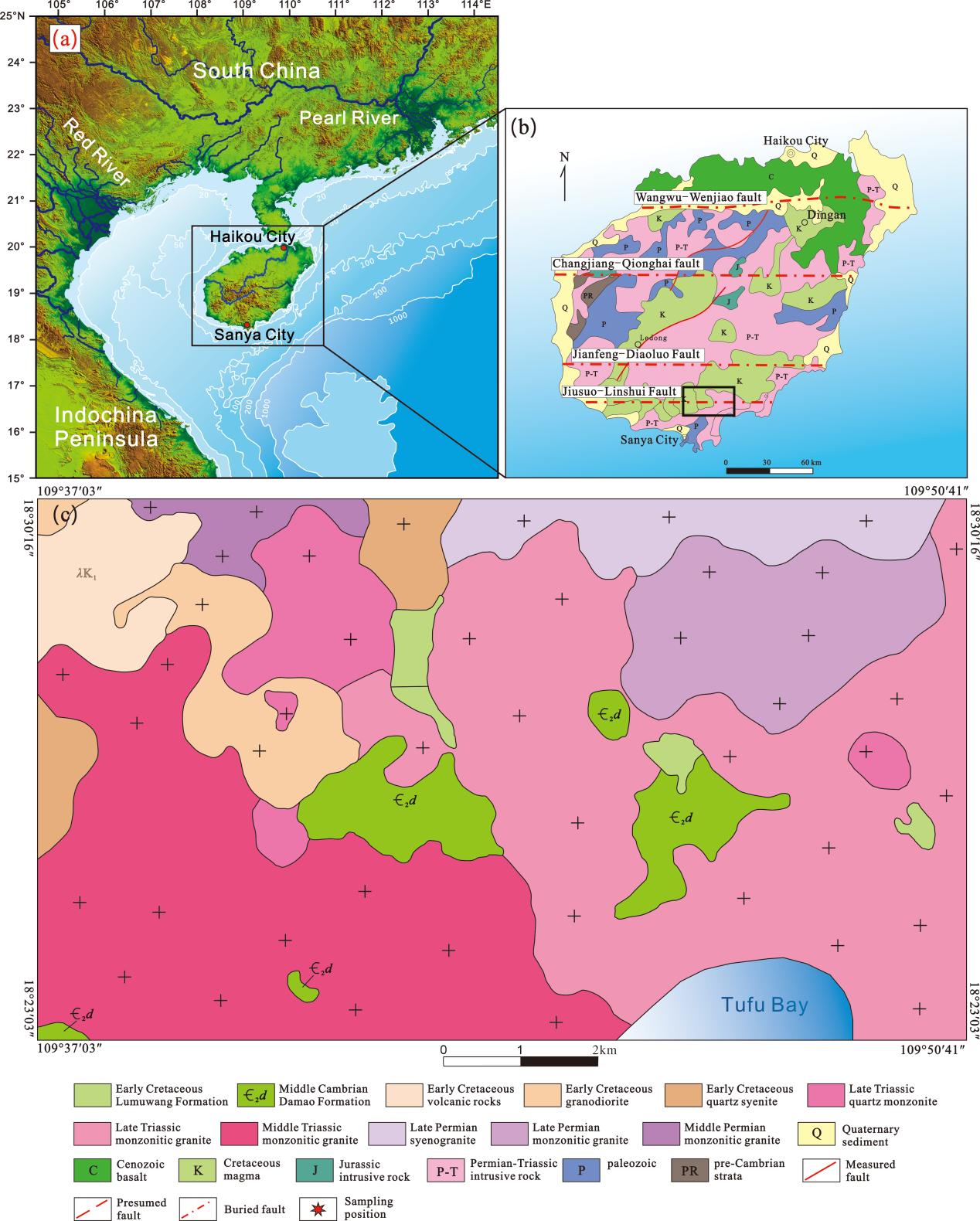


**Fig 1. Map of the Location of Hainan Island (a), The base map is adapted from Liang et al. [27], with modifications)、Geological Map of Hainan Island (b) and Study Area (c).**

The original basemap was created using the Digital Elevation Model (DEM). The public data of the DEM was obtained from the Geospatial Data Cloud (https://www.gscloud.cn/#page1) and was further processed using software CorelDRAW 2019 version.
