# Supplementary figures and images for: LA-ICP-MS Zircon U-Pb Ages, geochemical characteristics, and geological significance of the early cretaceous volcanic rocks in Haitangwan Town, Southern Hainan Island, China
Source: PLoS One. 2025 Dec 4;20(12):e0337464. doi: 10.1371/journal.pone.0337464 (PMC12677543; doi:10.1371/journal.pone.0337464)

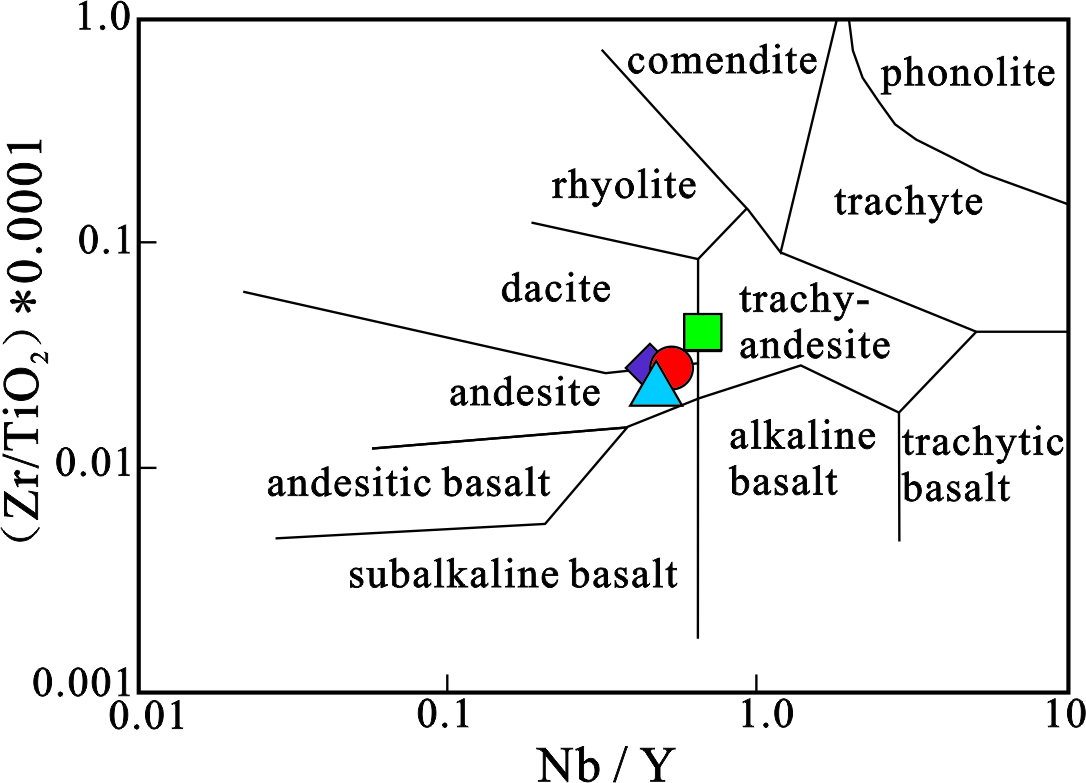


**Fig 2. Zr / TiO2-Nb / Y diagram of volcanicrocks（after Winchester and Floyd [29]）**

Supplement: S2 Fig — (DOCX) [file pone.0337464.s003.docx]

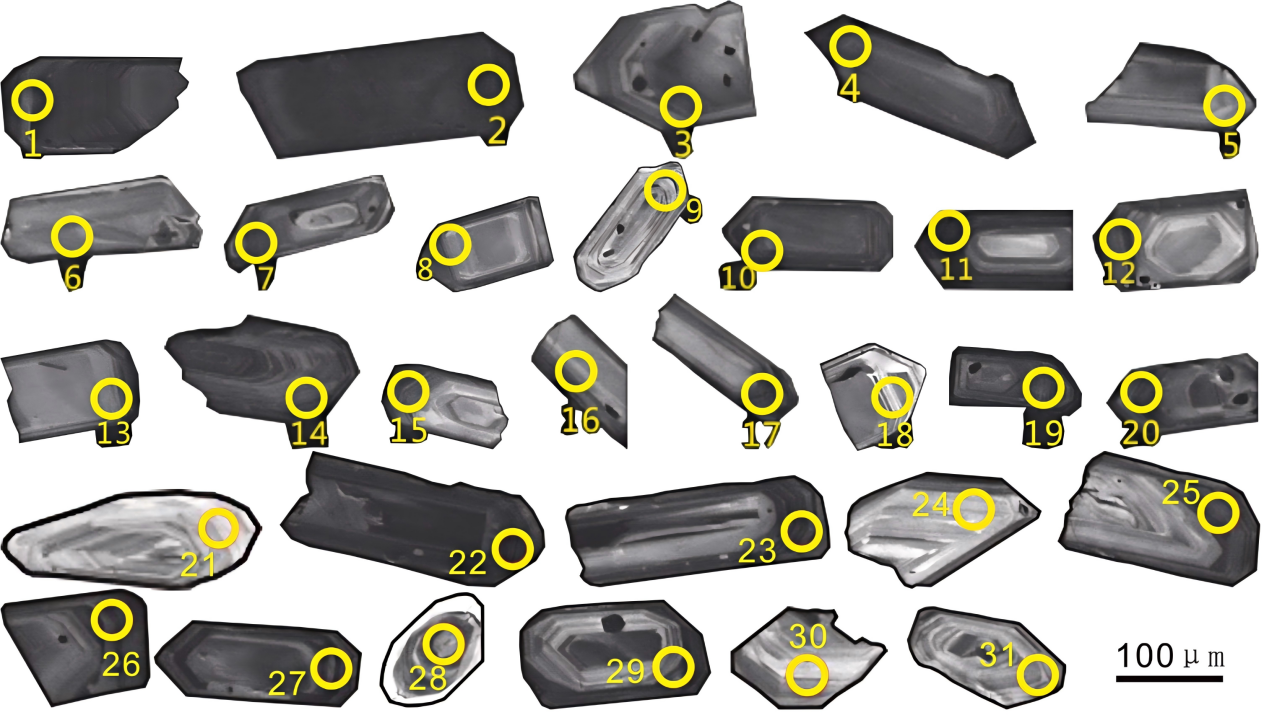


**Fig 4. Sample PMD05-15 partial zircon CL image**

Supplement: S4 Fig — (DOCX) [file pone.0337464.s005.docx]

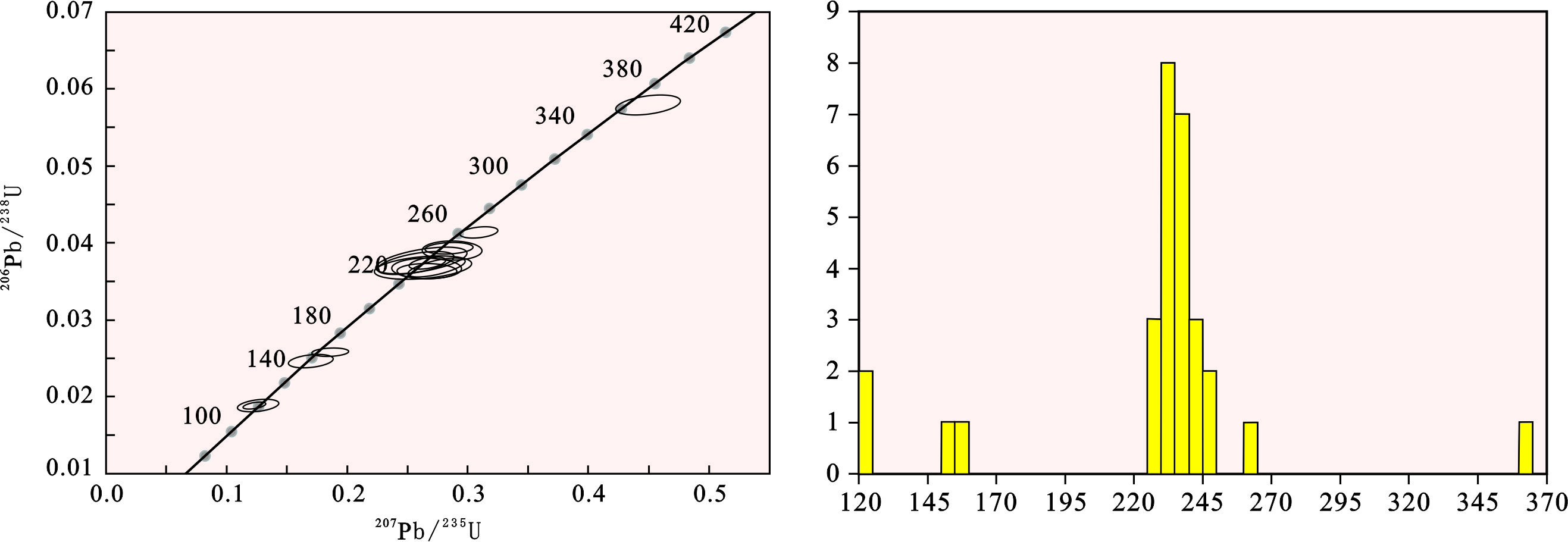


Fig 5. Concordia diagram and histogram of zircon U-Pb ages for sample PMD05-15

Supplement: S5 Fig — (DOCX) [file pone.0337464.s006.docx]

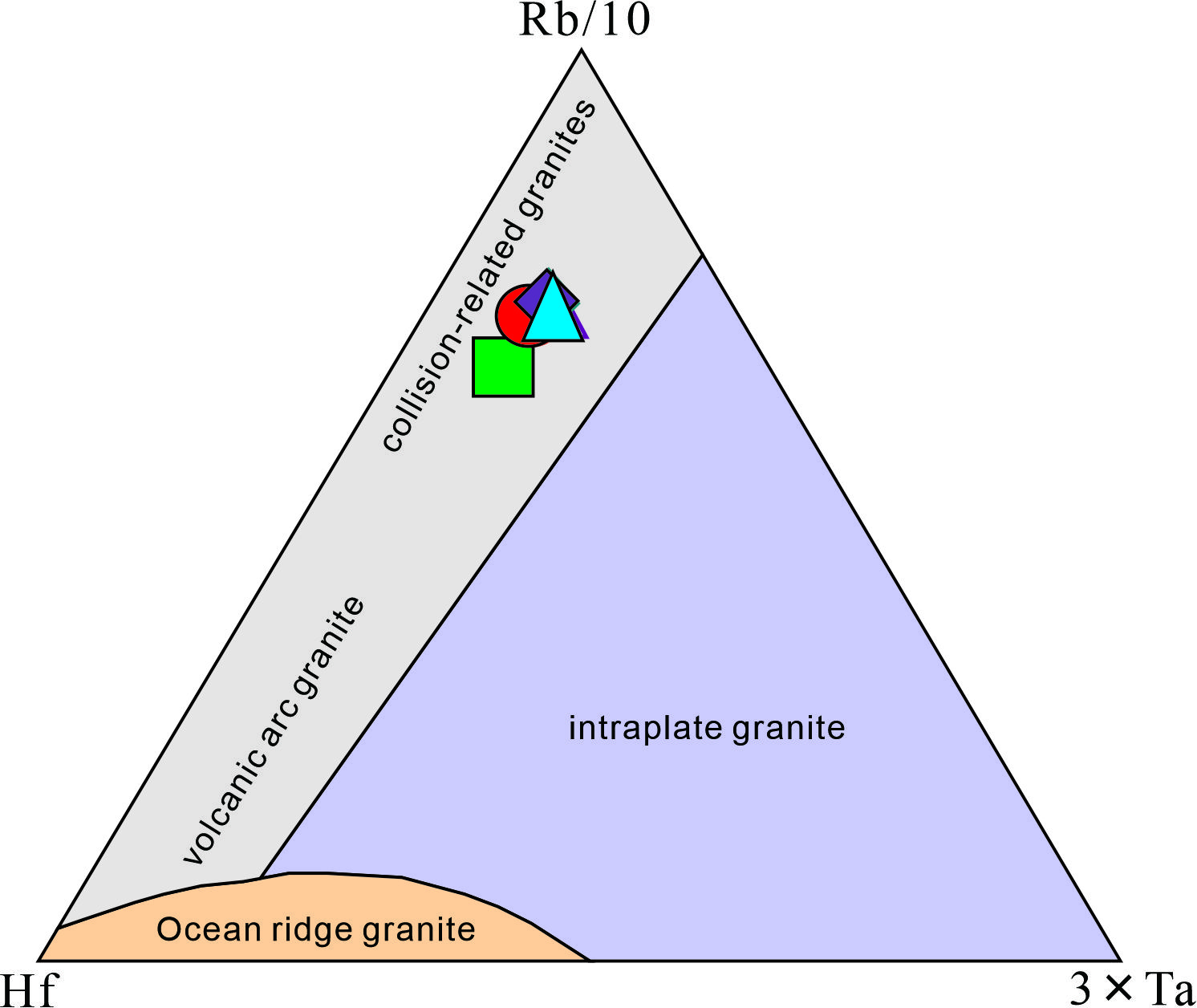


**Fig 10. Diagram of Early Cretaceous volcanic rocks Rb-Hf-Ta (after Harris et al. [66])**

Supplement: S10 Fig — (DOCX) [file pone.0337464.s011.docx]

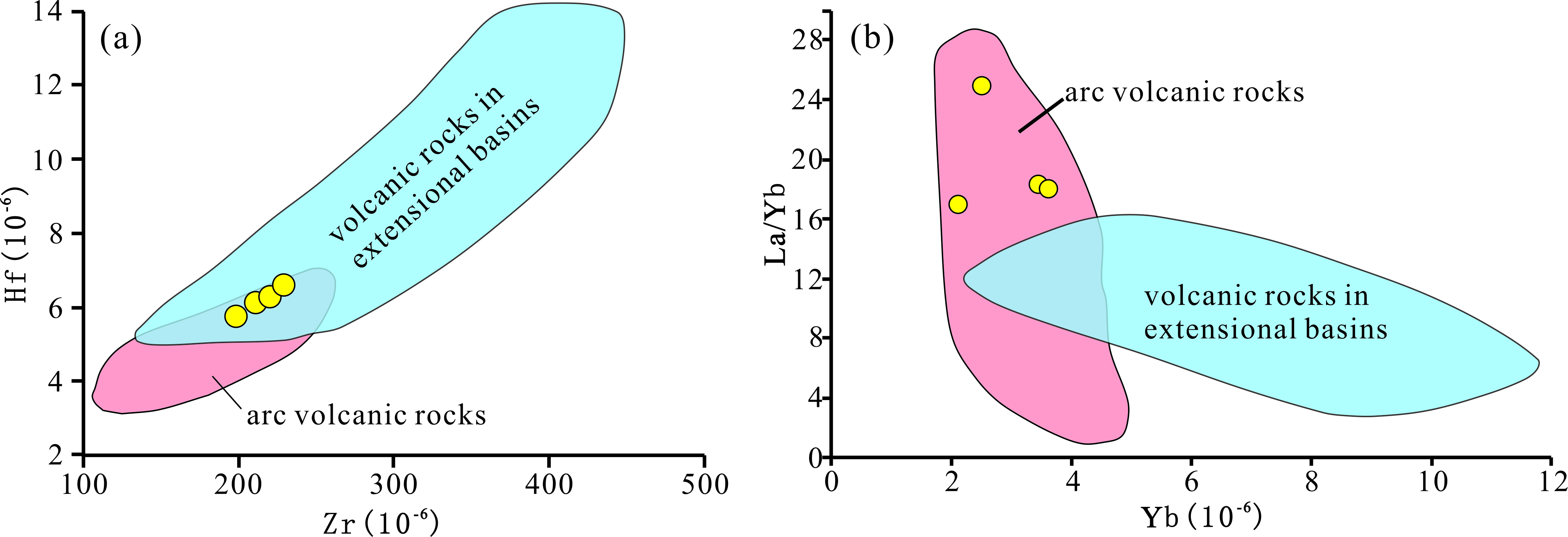


**Fig 12. Discriminant diagram of volcanic rock tectonic settings (after Condie [67])**

Supplement: S12 Fig — (DOCX) [file pone.0337464.s013.docx]

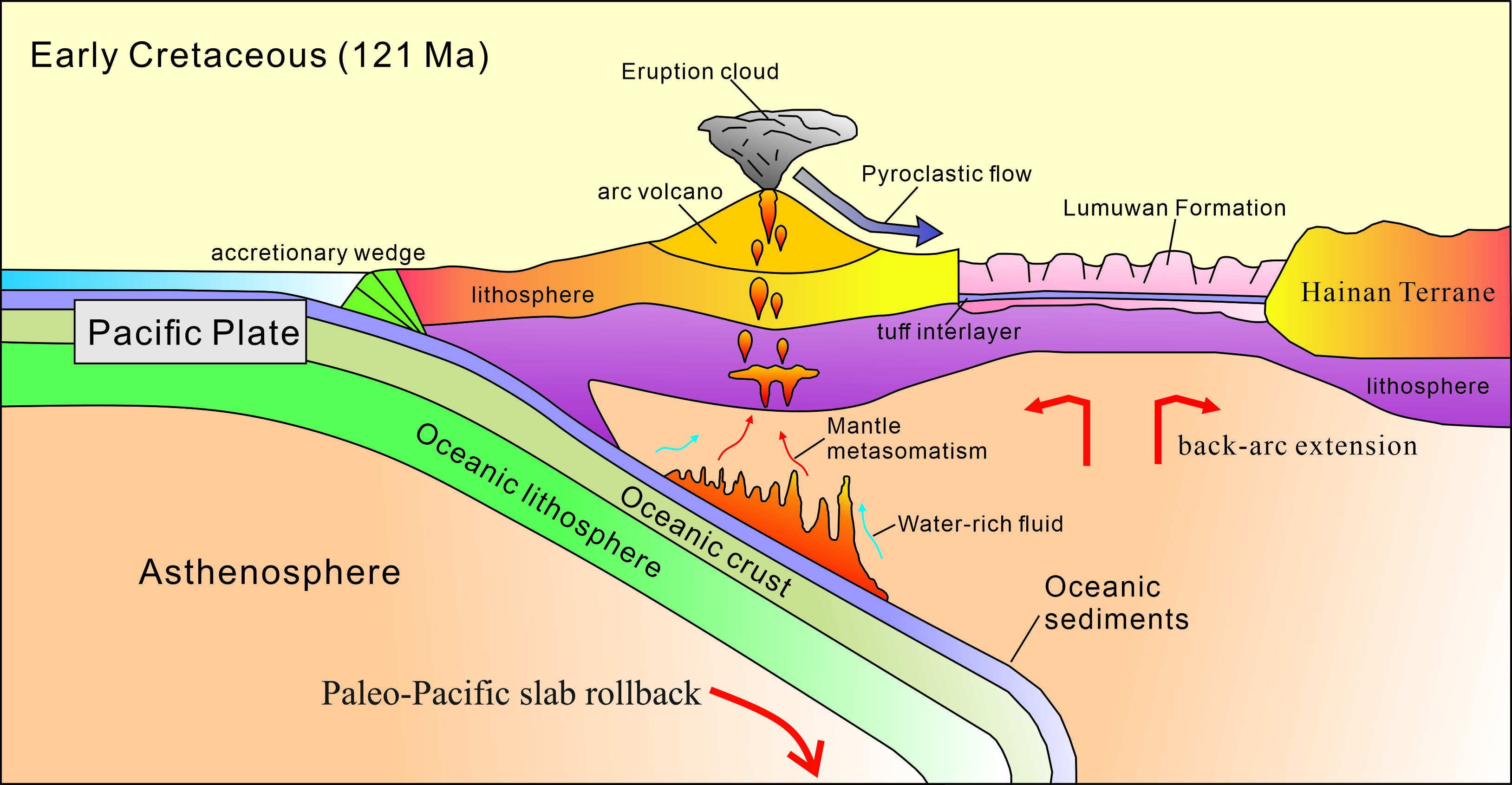


**Fig 13. Tectonic evolution model of the study area during the Early Cretaceous**

Supplement: S13 Fig — (DOCX) [file pone.0337464.s014.docx]
