# Supplementary material for: LA-ICP-MS Zircon U-Pb Ages, geochemical characteristics, and geological significance of the early cretaceous volcanic rocks in Haitangwan Town, Southern Hainan Island, China
Source: PLoS One. 2025 Dec 4;20(12):e0337464. doi: 10.1371/journal.pone.0337464 (PMC12677543; doi:10.1371/journal.pone.0337464)
Supplement: S3 Fig — (DOCX) [file pone.0337464.s004.docx]

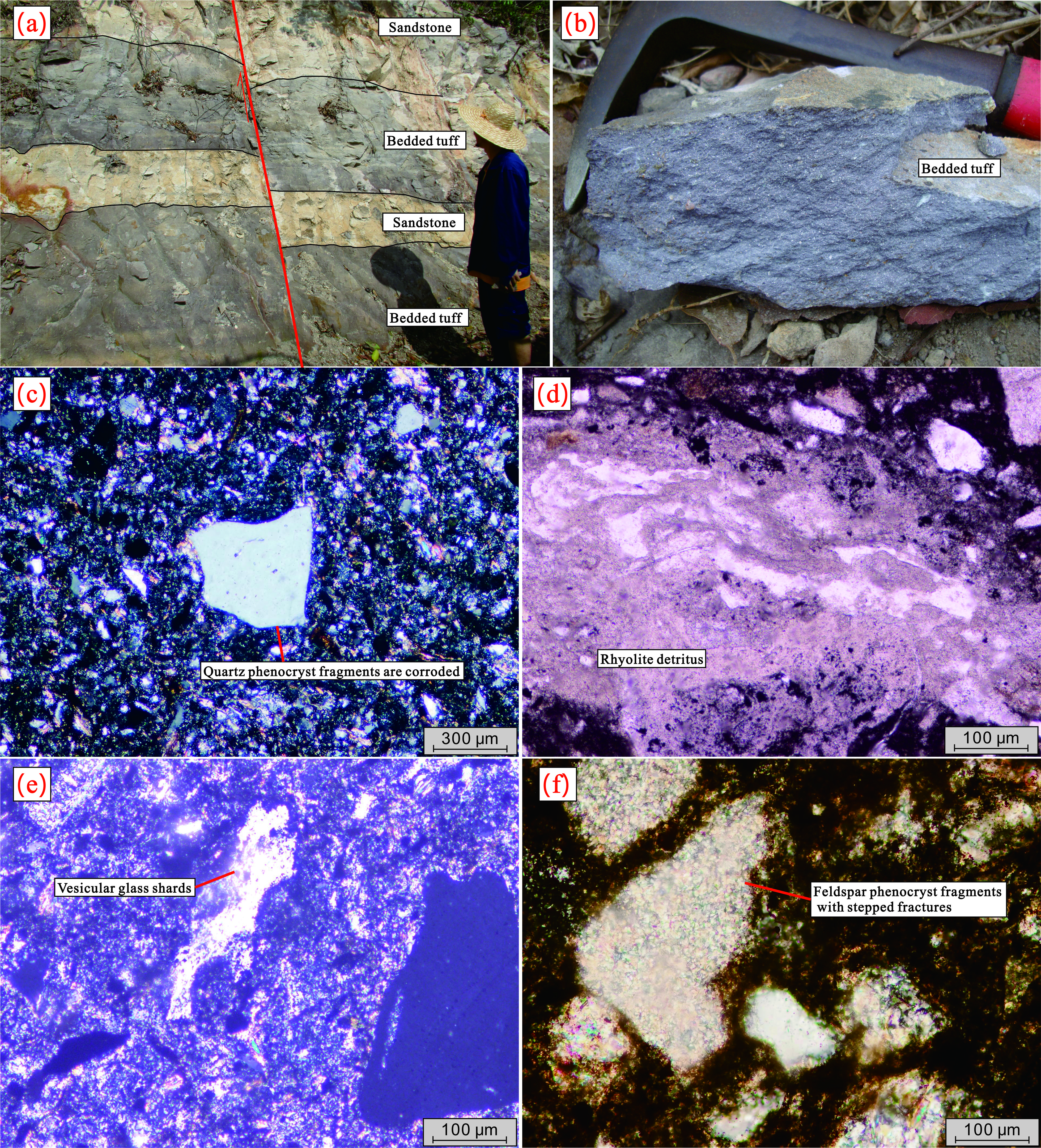


**Fig 3. Field and Microscopic Photographs of Tuff**

a Interbedded photograph of tuff and sandstone; b Close-up photograph of tuff specimen; c Photograph of corroded quartz phenocrysts, cross-polarized light (XPL); d Photograph of rhyolitic lithic fragments, plane-polarized light (PPL); e Photograph of volcanic glass shards, XPL; f Photograph of feldspar phenocrysts with conchoidal fracture, XPL.
