# Supplementary material for: LA-ICP-MS Zircon U-Pb Ages, geochemical characteristics, and geological significance of the early cretaceous volcanic rocks in Haitangwan Town, Southern Hainan Island, China
Source: PLoS One. 2025 Dec 4;20(12):e0337464. doi: 10.1371/journal.pone.0337464 (PMC12677543; doi:10.1371/journal.pone.0337464)
Supplement: S6 Fig — R diagram and SiO2-K2O diagram. (DOCX) [file pone.0337464.s007.docx]

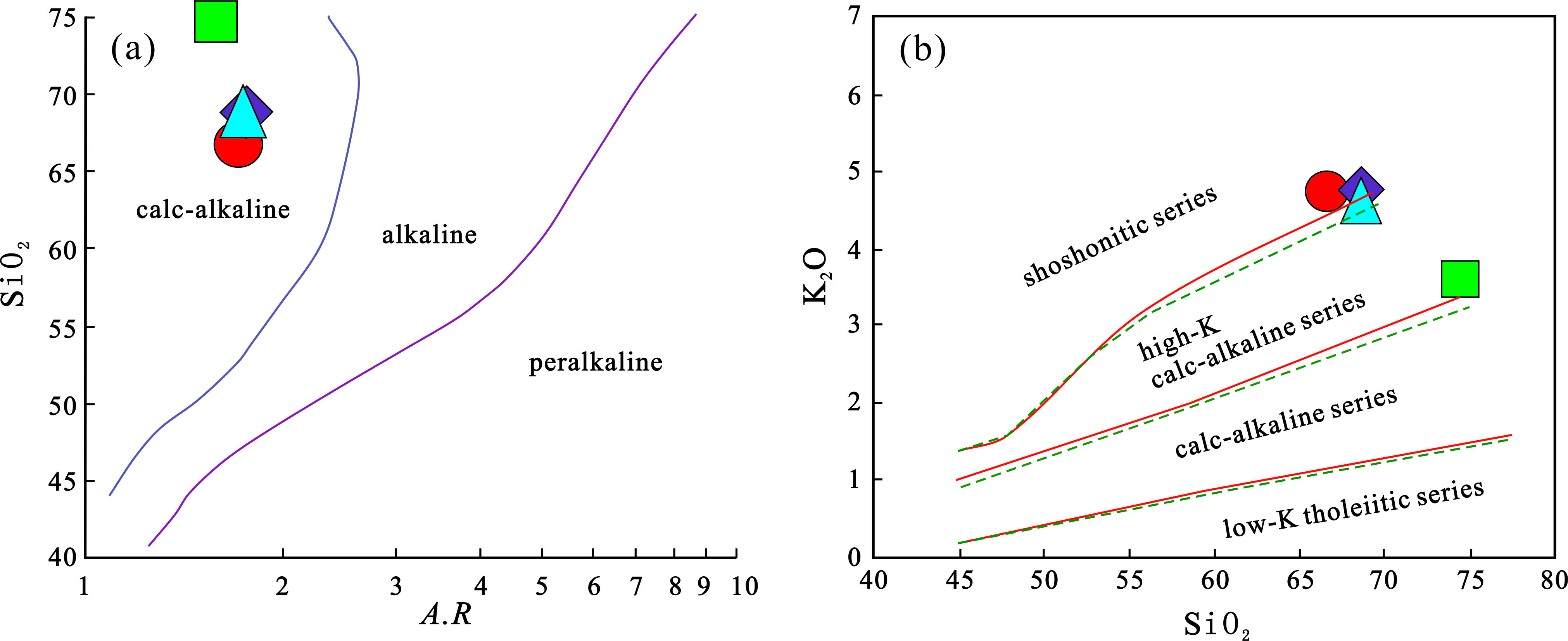


**Fig 6. SiO_2_-A.R diagram (after Wright [35]) and SiO_2_-K_2_O diagram (after Rickwood [36]) for volcanic rocks in the study area**
