# Supplementary material for: LA-ICP-MS Zircon U-Pb Ages, geochemical characteristics, and geological significance of the early cretaceous volcanic rocks in Haitangwan Town, Southern Hainan Island, China
Source: PLoS One. 2025 Dec 4;20(12):e0337464. doi: 10.1371/journal.pone.0337464 (PMC12677543; doi:10.1371/journal.pone.0337464)
Supplement: S7 Fig — (DOCX) [file pone.0337464.s008.docx]

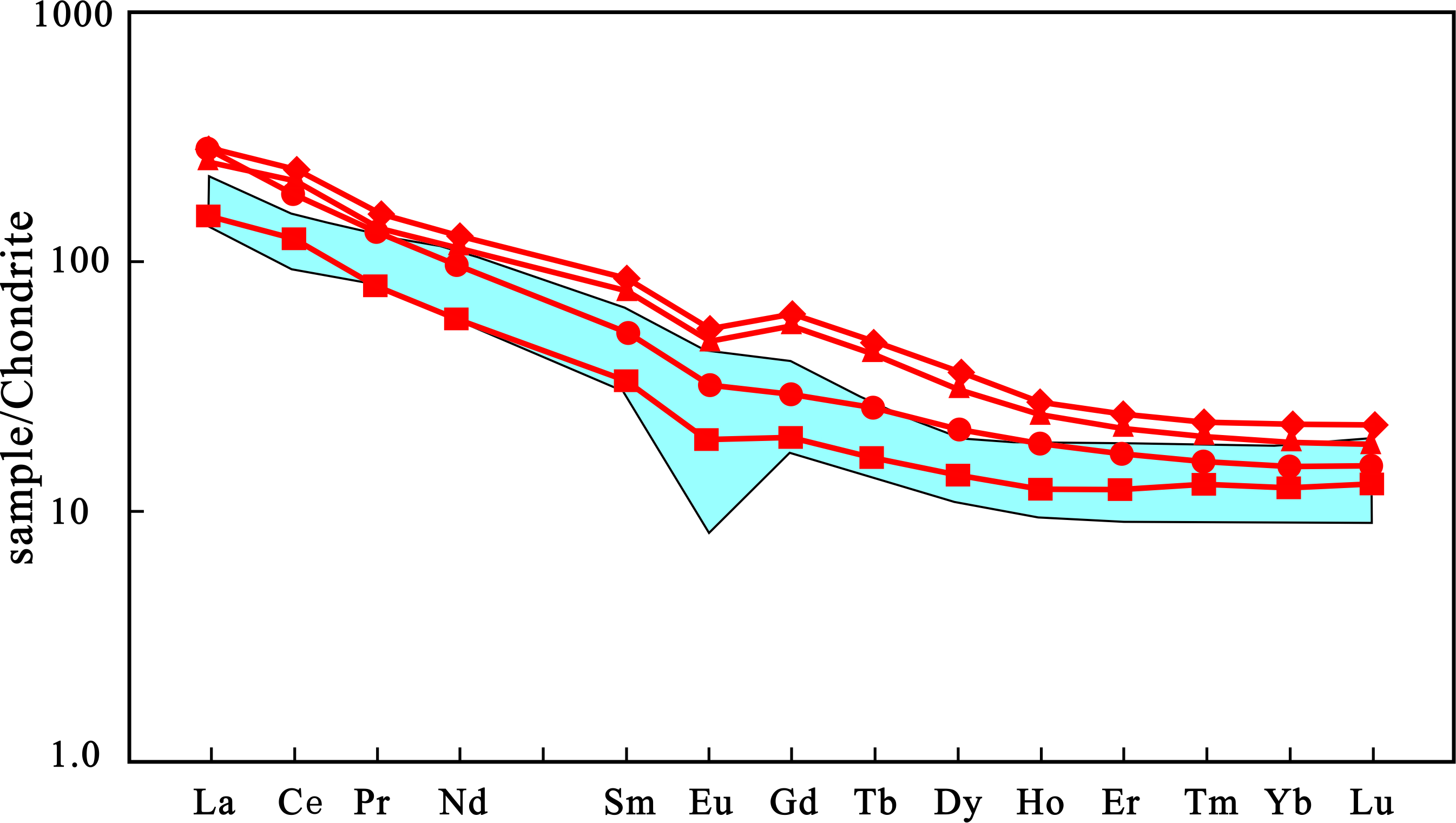


**Fig 7. Chondrite-normalized REE distribution pattern diagram of volcanic rocks in the study area**

(Shaded areas represent the background values of Mesozoic volcanic rocks in Hainan Island [13]; chondrite standard values after Sun and McDonough [39])
