# Supplementary material for: LA-ICP-MS Zircon U-Pb Ages, geochemical characteristics, and geological significance of the early cretaceous volcanic rocks in Haitangwan Town, Southern Hainan Island, China
Source: PLoS One. 2025 Dec 4;20(12):e0337464. doi: 10.1371/journal.pone.0337464 (PMC12677543; doi:10.1371/journal.pone.0337464)
Supplement: S9 Fig — (DOCX) [file pone.0337464.s010.docx]

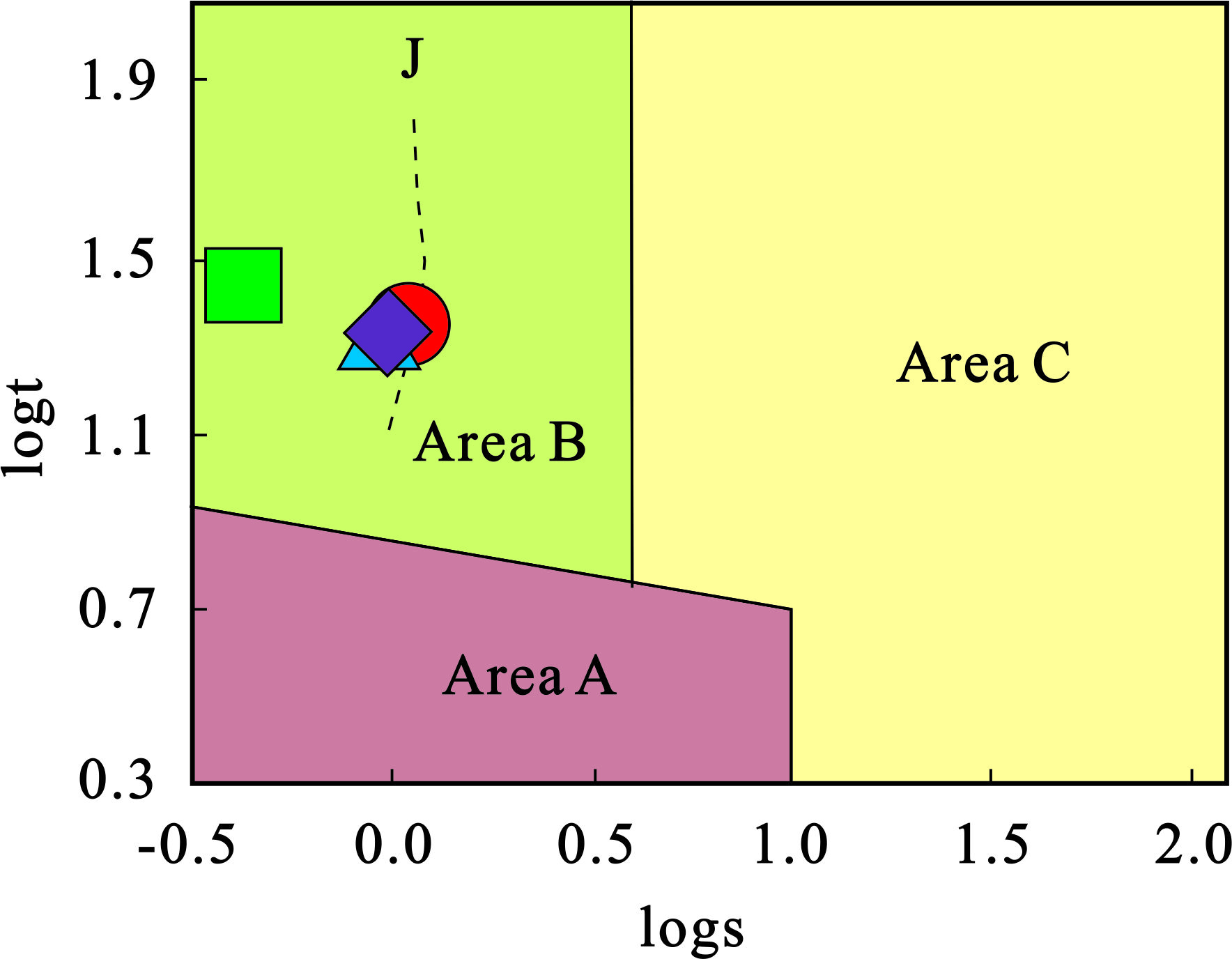


**Fig 9. logt-logs diagram of volcanic rocks (after Gottini [55])**

Area A: Volcanic rocks from non-tectonic belts (stable intraplate tectonic regions); Area B: Volcanic rocks from orogenic belts (island arcs and active continental margin regions); Area C: Alkaline rocks derived from volcanic rocks in Areas A and B.
