# Supplementary material for: LA-ICP-MS Zircon U-Pb Ages, geochemical characteristics, and geological significance of the early cretaceous volcanic rocks in Haitangwan Town, Southern Hainan Island, China
Source: PLoS One. 2025 Dec 4;20(12):e0337464. doi: 10.1371/journal.pone.0337464 (PMC12677543; doi:10.1371/journal.pone.0337464)
Supplement: S11 Fig — (DOCX) [file pone.0337464.s012.docx]

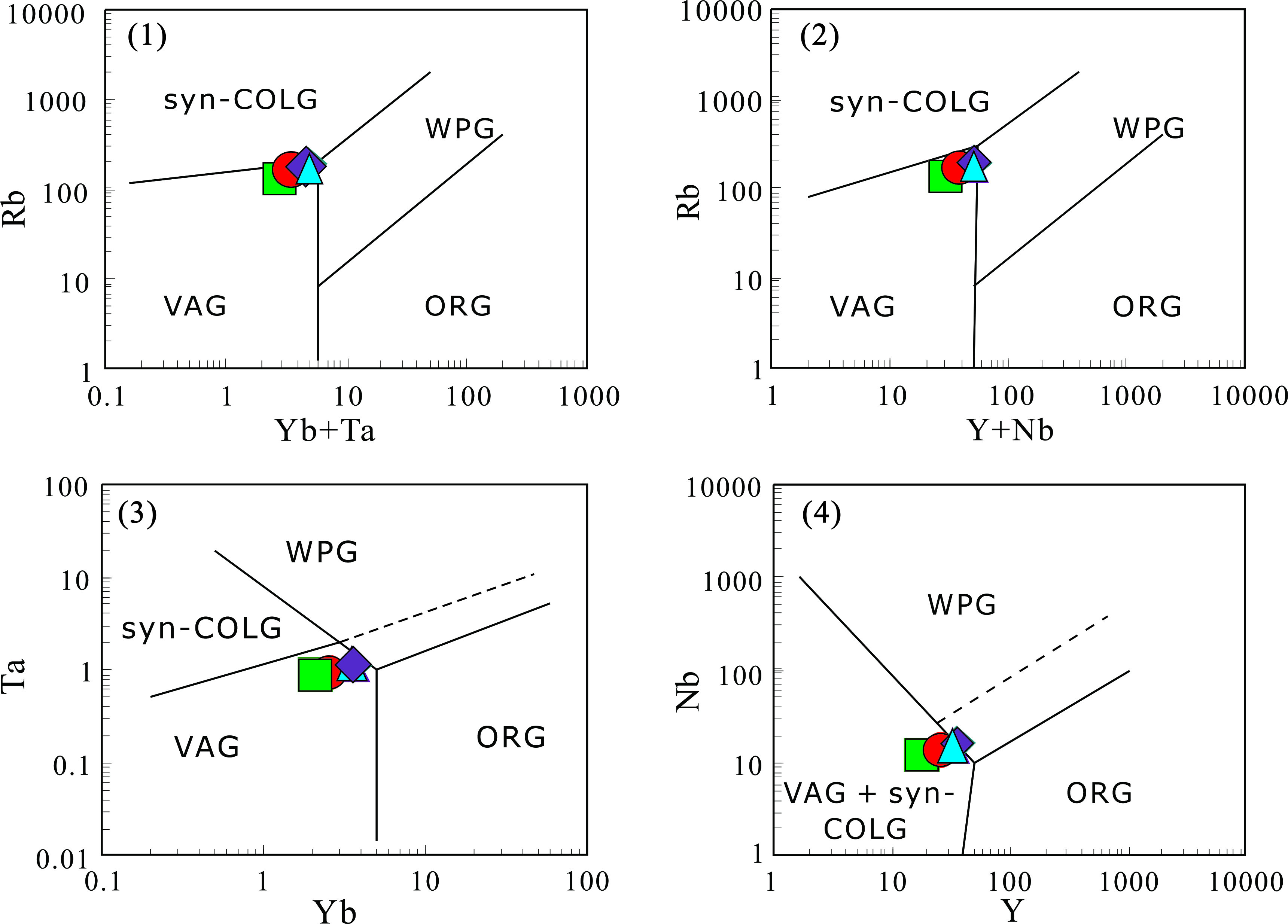


**Fig 11. Discriminant diagram of volcanic rocks based on immobile elements (after Pearce et al. [56])**

VAG: Volcanic arc granite; WPG: Within-plate granite; S-COLG: Syn-collisional granite; ORG: Ocean ridge granite; A-ORG: Anomalous ocean ridge granite
